# Dynamics of human milk oligosaccharides in early lactation and relation with growth and appetitive traits of Filipino breastfed infants

**DOI:** 10.1038/s41598-022-22244-7

**Published:** 2022-10-15

**Authors:** Tinu M. Samuel, Mickaël Hartweg, Jowena D. Lebumfacil, Katherine. B. Buluran, Rachel. B. Lawenko, Elvira M. Estorninos, Aristea Binia, Norbert Sprenger

**Affiliations:** 1 Nestlé Product Technology Center-Nutrition, Société des Produits Nestlé S.A., 1800 Vevey, Switzerland; 2 Clinical Research Unit, Société des Produits Nestlé SA, 1000 Lausanne, Switzerland; 3 Clinical Research Operations, Wyeth Philippines, Inc., Makati City, 1200 Philippines; 4 Asian Hospital & Medical Center, Muntinlupa City, 1780 Philippines; 5 Nestlé Institute of Health Sciences, Société des Produits Nestlé SA, 1000 Lausanne, Switzerland

**Keywords:** Health care, Paediatric research, Medical research, Paediatric research

## Abstract

Human milk oligosaccharides play a key role in the maturation of the infant gut microbiome and immune system and are hypothesized to affect growth. This study examined the temporal changes of 24 HMOs and their associations to infant growth and appetitive traits in an exploratory, prospective, observational, study of 41 Filipino mother-infant dyads. Exclusively breastfed, healthy, term infants were enrolled at 21–26 days of age (≈ 0.75 mo) and followed for 6 months. Infant growth measures and appetitive traits were collected at visit 1 (V1) (≈ 0.75 mo), V2 (≈ 1.5 mo), V3 (2.5 mo), V4 (2.75 mo), V5 (4 mo), and V6 (6 mo), while HMOs were measured at V1, V2, V3 and V5. Overall exposure to each HMO was summarized as area under the curve from baseline to 4 months of age and examined in association with each measure of growth at 6 months using linear regression adjusted for maternal age at birth, infant sex, birth weight, and mode of delivery. We saw modest associations between several HMOs and infant growth parameters. Our results suggest that specific HMOs, partly as proxy for milk groups (defined by Secretor and Lewis status), may be associated with head circumference and length, increasing their relevance especially in populations at the lower end of the WHO growth curve. We did not identify the same HMOs associated with infant appetitive traits, indicating that at least in our cohort, changes in appetite were not driving the observed associations between HMOs and growth.

Clinical trial registration: NCT03387124.

## Introduction

Human milk (HM) is the globally accepted ideal source of nutrition for infants, providing a mixture of nutrients and bioactive components that support appropriate growth, good digestibility and tolerance, as well as contributing additional benefits to the development of the immune system and a healthy gut microbiota^1,2^. While many short and long-term benefits of longer duration of breastfeeding, including lower infectious morbidity and mortality among infants, lower rates of infant dental malocclusions, and positive cognitive outcomes are well-established^3,4^, the association between breastfeeding and risk of later childhood overweight and obesity, although probable, is less conclusive^3,5–7^. The existing literature is hampered by significant heterogeneity between studies (for example, in the definitions of breastfeeding exclusivity and duration, as well as the timing and measurement processes for measures of adiposity) and the potential for residual confounding^3,8–11^. The mechanistic pathways that may link breastfeeding to protection against childhood obesity are not yet well understood^12^, but a variety of HM components, including macro- and micronutrients as well as cytokines and hormones, likely contribute to variations in growth^13,14^. Further, breastfeeding may directly affect feeding preferences in a manner that prevents obesogenic eating patterns^12^, and additionally, behavioral aspects of breastfeeding may help infant-mother dyads with self-regulation and attentiveness to fullness and satiety cues^14^. Collectively, these effects may contribute to preventing excess or rapid weight gain in breastfed infants, leading to reduced risks of later overweight and obesity^15–17^.

A large group of structurally diverse oligosaccharides, known collectively as human milk oligosaccharides (HMOs), which represent the third largest solid component of HM after lactose and lipids^18,19^ have increasingly gained attention in relation to infant growth^20–28^. The quantity and composition of HMOs in HM varies greatly between women and within the same woman by stage of lactation^29,30^. The fucosyltransferases (FUT) 2 and 3 encoded by the Secretor (Se) and Lewis (Le) gene, respectively, are the primary enzymes involved in the synthesis of the fucosylated HMOs^31^. These represent the largest family of HMOs. Maternal genetic polymorphism for the Se and Le genes explains to a large extent the HMO variability between mothers^31^ Most importantly, maternal Se and Le status with presence or absence of functional FUT2 and/or FUT3 enzymes allows definition of four milk groups with distinct HMO profiles^31,32^. Yet, additional maternal conditions like pre-pregnancy BMI, maternal age, gestational age, mode of delivery and parity are thought to also contribute to interindividual HMO variability^30,33–35^.

A currently debated question is whether HMO variability in HM influences infant growth and development. In addition to their role in positively affecting the composition of the gut microbiome^18^ and their ability to alter immune responses^18^, HMOs are also hypothesized to affect growth via multiple plausible mechanisms. These include functional gut maturation, reduced intestinal infection and inflammation allowing for better absorption of nutrients^36^ and influencing appetite regulation via microbial metabolites such as short chain fatty acids^37^. However, systemic, as yet unidentified effects cannot be excluded as HMOs pass into the circulatory system^38,39^. Several studies have examined HMOs in relation to the growth of breastfed infants with consistent findings being reported only in a few^20–28,35^. While the microbiome is thought to be a key mechanism linking HMOs to infant growth, appetite may also play a role as proposed in one recent report that found significant positive and negative associations between specific HMOs and food responsiveness, which reflects a measure of an infant’s drive to eat^40^ and can potentially impact infant weight gain. Most studies were conducted in populations with European ancestry with only one examining associations between HMOs and infant growth in an Asian population from Singapore^28^. Based on these gaps in the existing literature, there is a need for further studies to examine the role of specific HMOs in infant growth in diverse populations. Therefore, the objective of this exploratory study was to examine the temporal change of 24 different HMOs as well as associations between these HMOs and infant growth and appetitive traits in a cohort of exclusively breastfed Filipino infants.

## Results

### Study population

**Figure 1:**
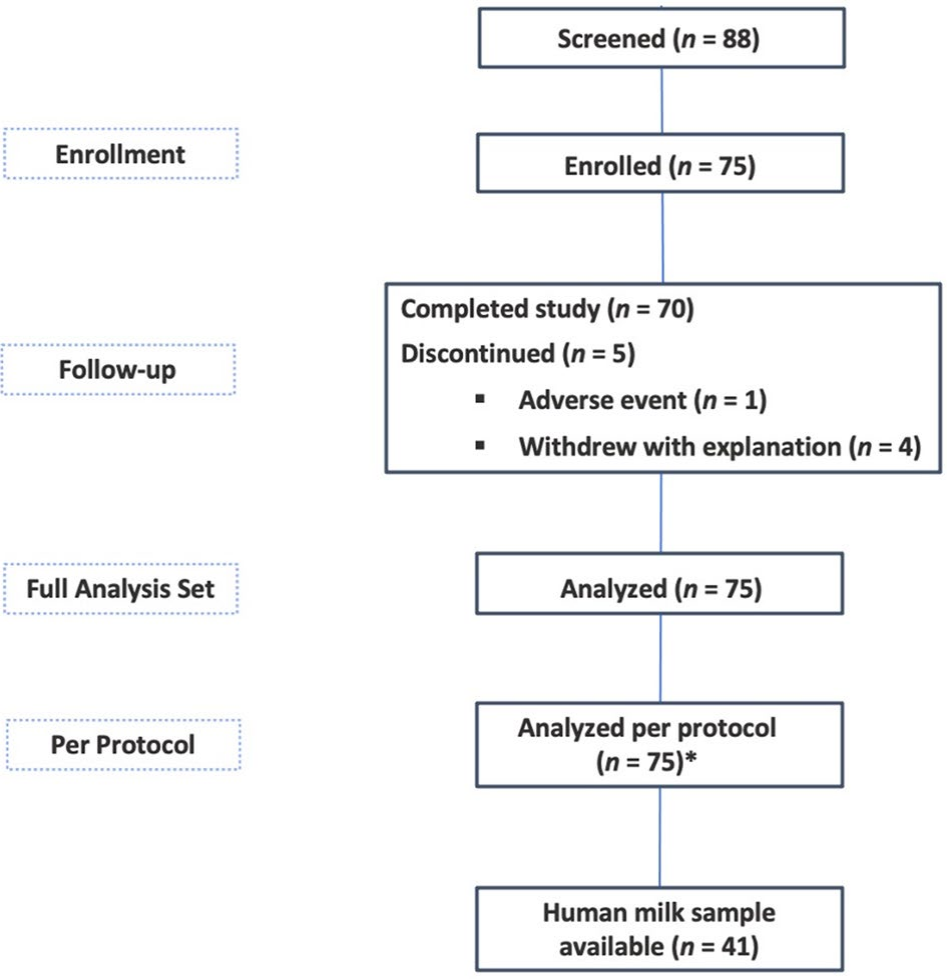
Study design and subject flow chart. *Only 1 enrolled subject had major protocol deviation (“Informed consent for breastmilk sample is signed but breastmilk sample is not collected”). This subject was not excluded from the per-protocol set for this deviation and thus the full analytic set and per protocol datasets both include 75 subjects.

Eighty-eight infants were screened for eligibility and 75 enrolled in the study based on a calculated sample size for the primary study objective related to stool consistency. Forty-one mothers provided HM samples and these infant-mother dyads make up the analytic population for this study (Fig. 1). Characteristics of the 41 infant-mother pairs are shown in Table 1. The infants were born at an average of 38.8 weeks gestational age and 51.2% were female. Average age at enrollment was 23.9 days. Most infants were delivered vaginally (87.8%), and mothers were on average 26.3 years old at time of delivery. Baseline characteristics of the infants and mothers by milk group are shown in Supplemental Table 1. The largest proportion of infants were in the Se+/Le+ group (N = 25), followed by Se−/Le+ (N = 10), Se+ /Le− (N = 5) and Se−/Le− (N = 1). Hence, 73% of mothers showed a Se+ and 27% a Se− phenotype.

**Table 1:** Infant and maternal characteristics (N = 41).

	Mean [SD] or N (%)	Median [25th, 75th percentiles]
**Infant Characteristics**		
Sex		
Female	21 (51.2%)	
Male	20 (48.8%)	
Gestational age at birth (weeks)	38.8 [1.4]	39 [38, 40]
Age at enrollment (days)	23.9 [1.9]	24 [22, 26]
Birth weight, grams	3082.2 [310.6]	3040 [2820, 3330]
Birth length, cm	50.0 [2.0]	50 [48, 51]
Weight at enrollment [21–26 days], grams	3884.2 [402.3]	3950 [3593, 4110]
Length at enrollment [21–26 days], cm	50.9 [1.6]	50.6 [49.6, 51.8]
BMI at enrollment [21–26 days], kg/m^2^	15.0 [1.2]	15.1 [14.1, 15.7]
Head circumference at enrollment [21–26 days], grams	35.9 [0.9]	35.9 [35.0, 36.6]
**Maternal characteristics**		
Delivery type		
Vaginal	36 (87.8%)	
Caesarean	5 (12.2%)	
Maternal age at birth, years	26.3 [5.4]	27 [22, 28]
Parity	2.6 [1.4]	2 [2, 3]

### HMO trajectories

**Figure 2:**
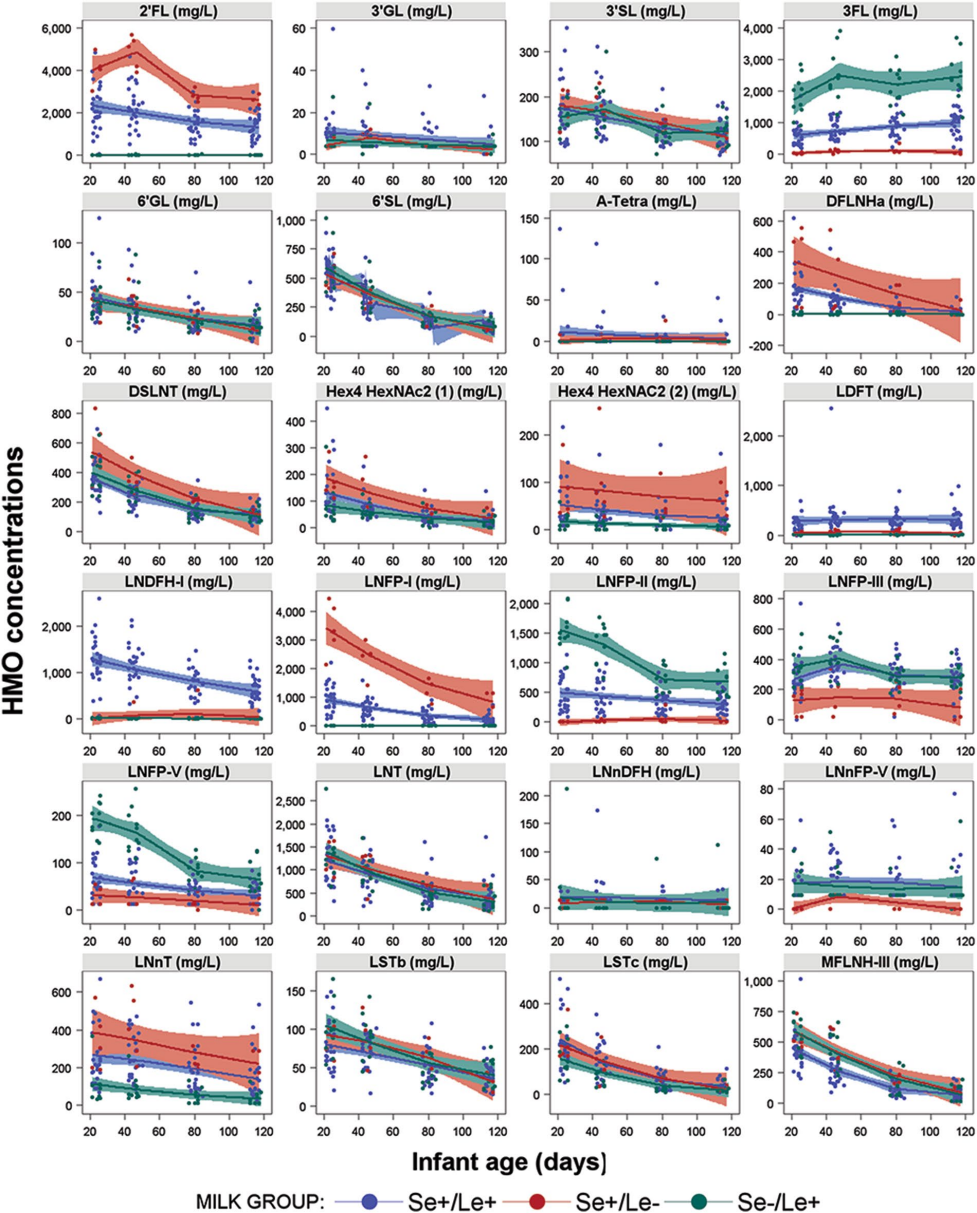
Trajectories of HMO concentrations over the first 4 months of lactation by milk group with n = 25 for Se+/Le+, n = 5 for Se+/Le−, n = 10 for Se−/Le+ . Not shown is n = 1 for Se−/Le−. Full names of all HMOs are provided in the methods section.

Twenty-four HMOs were quantified from ≈ 0.75 through 4 months of infant age and their trajectories are shown within strata of Se/Le milk groups, except for the n = 1 Se−/Le−, in Fig. 2. Summary statistics of the 24 quantified HMOs by time of lactation are provided in Supplemental Table 2 and a Pearson correlation matrix between the measured HMOs is shown in Supplemental Fig. 1. For most HMOs, the concentration numerically decreased over time of lactation from infant age ≈ 0.75 through 4 months. A few HMOs had relatively flat concentrations over the study period including A-tetra, LDFT, and LNnDFH. The galacto-oligosaccharide 3’GL was barely detectable, while 6’GL was slightly higher and decreased with time. 3FL slightly increased over the observed period. Apparent differences in the HMO profile and concentrations exist between milk groups (Fig. 2). Compared to the other milk groups, milk group Se+/Le+ generally showed an intermediate concentration of 2’FL, 3FL, DFLNHa, LNFP-I, -II, -V and LNnT, a higher concentration of LDFT and LNDFH-I and a lower concentration of MFLNH-III. Higher concentrations of 2’FL, DFLNHa, DSLNT, LNFP-I and LNnT were seen in the Se+ /Le− milk group together with lower concentrations of 3FL, LNFP-III, -V and LNnFP-V compared to the other 2 milk groups. Lastly, the Se−/Le+ milk group showed highest concentrations of 3FL, LNFP-II and LNFP-V and lowest of LNnT.

### Infant growth

Detailed anthropometric measurements at each study visit (weight, length, head circumference (HC), BMI, Weight-for-age z-score, Weight-for-length z-score, Length-for-age z-score, BMI-for-age z-score, HC-for-age z-score, weight gain/day and length gain/week) by infant sex are provided in Supplemental Table 3 together with box plots in Supplemental Figs. 2 and 3. As expected, boys had higher weights, lengths, and head circumferences at all timepoints compared to girls. In this cohort of Filipino infants, median z-scores as well as the 25th and 75th percentiles for weight-for-age, length-for-age, head circumference-for-age, BMI-for-age, and weight-for-length were all within WHO reference ranges (± 2 Standard Deviations (SD)) for both boys and girls. However, particularly for length, median values in this population were overall lower than the WHO reference medians. Examining patterns over time, growth generally increased more rapidly at younger ages before slowing down by 6 months (Supplemental Fig. 3).

**Figure 3:**
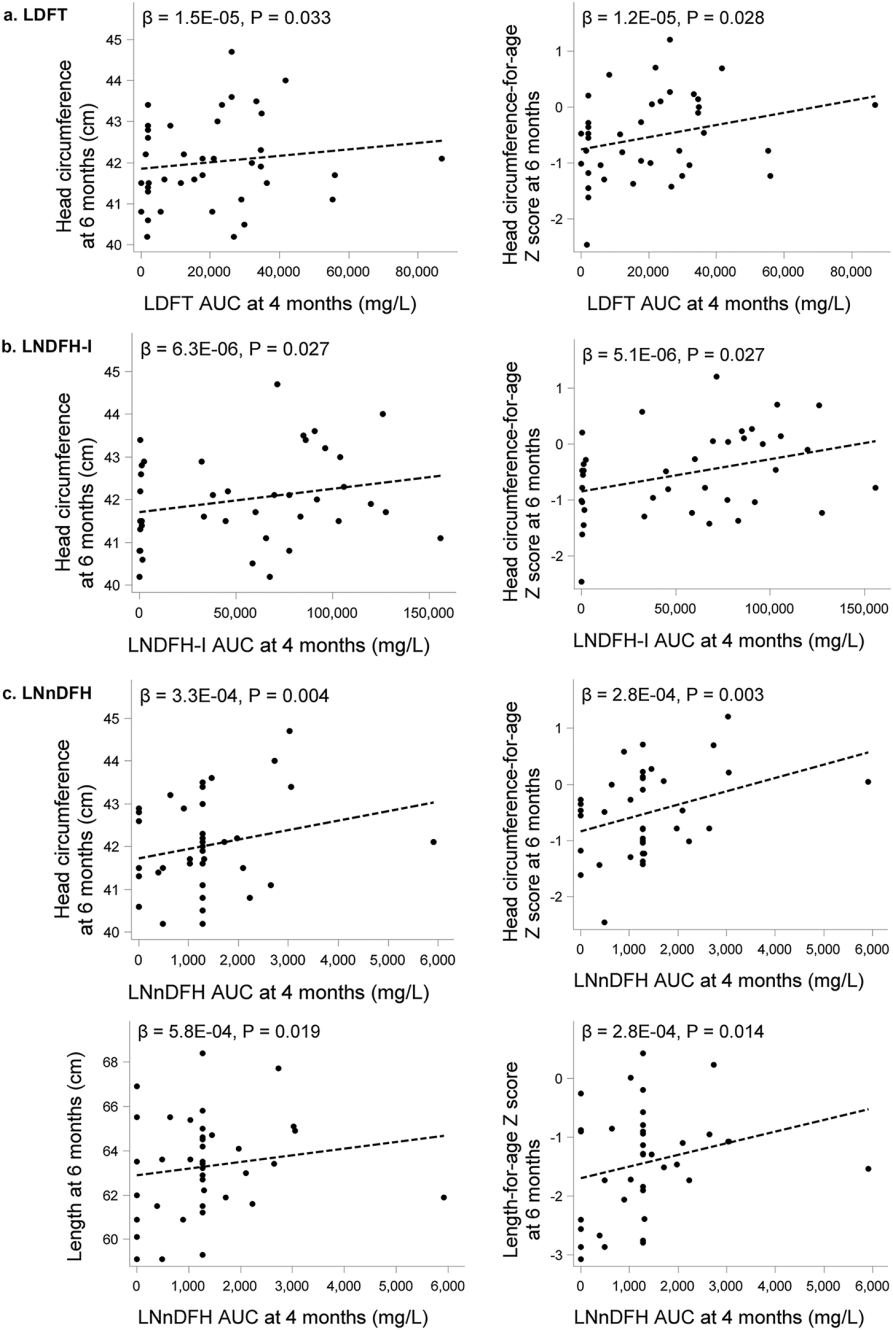

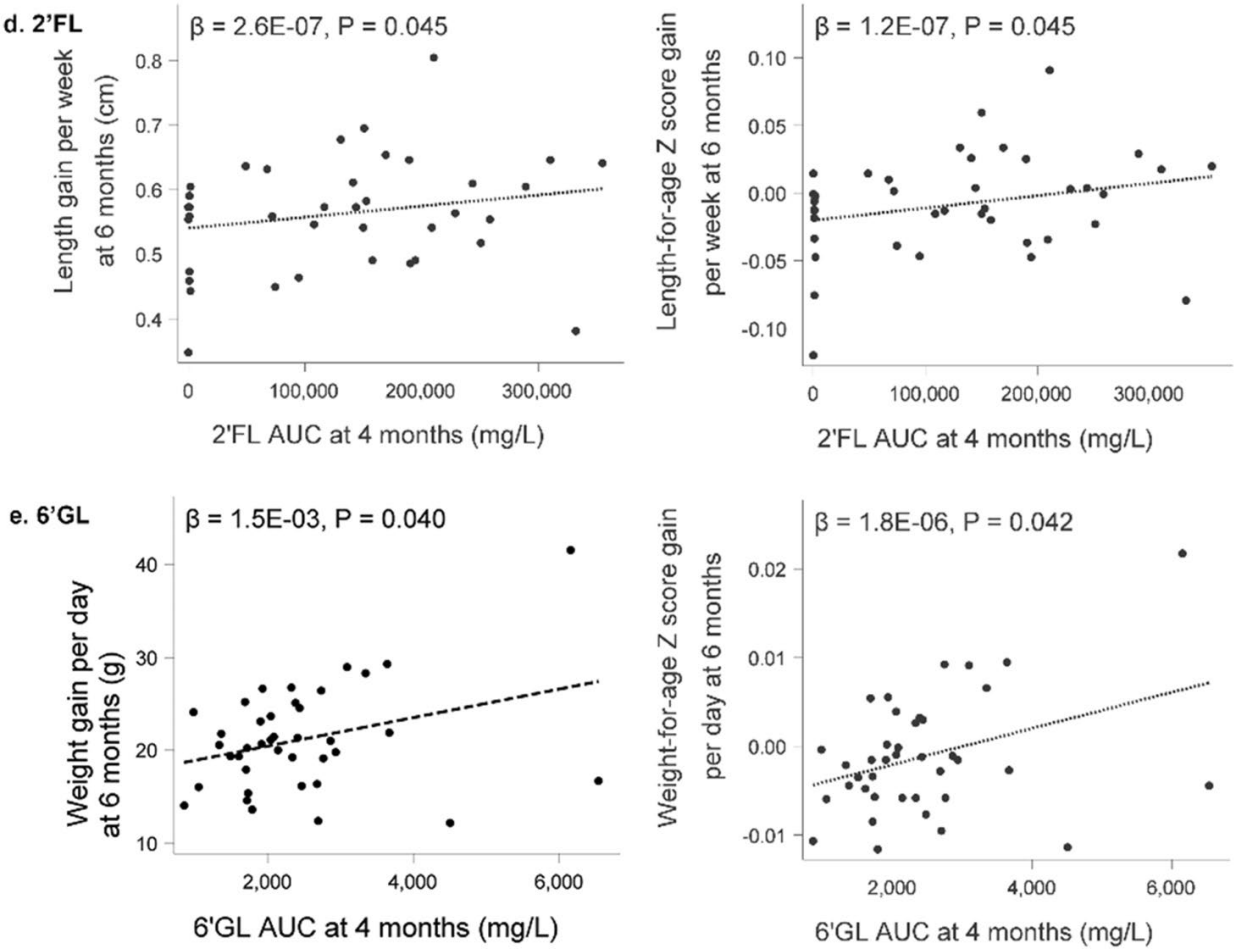
Scatterplots for select HMO Area Under the Curve (AUC) until 4 months of lactation and head circumference and length at 6 months of age (**a**–**c**) and length- and weight gain, respectively (**d**,**e**) from birth to 6 months of age (N = 41). LDFT, lacto-difucosyltetraose; LNDFH-I, lacto-N-difucosylhexaose-I; LNnDFH, lacto-N-neodifucohexaose; 2’FL, 2’fucosyllactose; 6’GL, 6’galactosyllactose.

### Association between HMOs and growth

**Figure 4:**
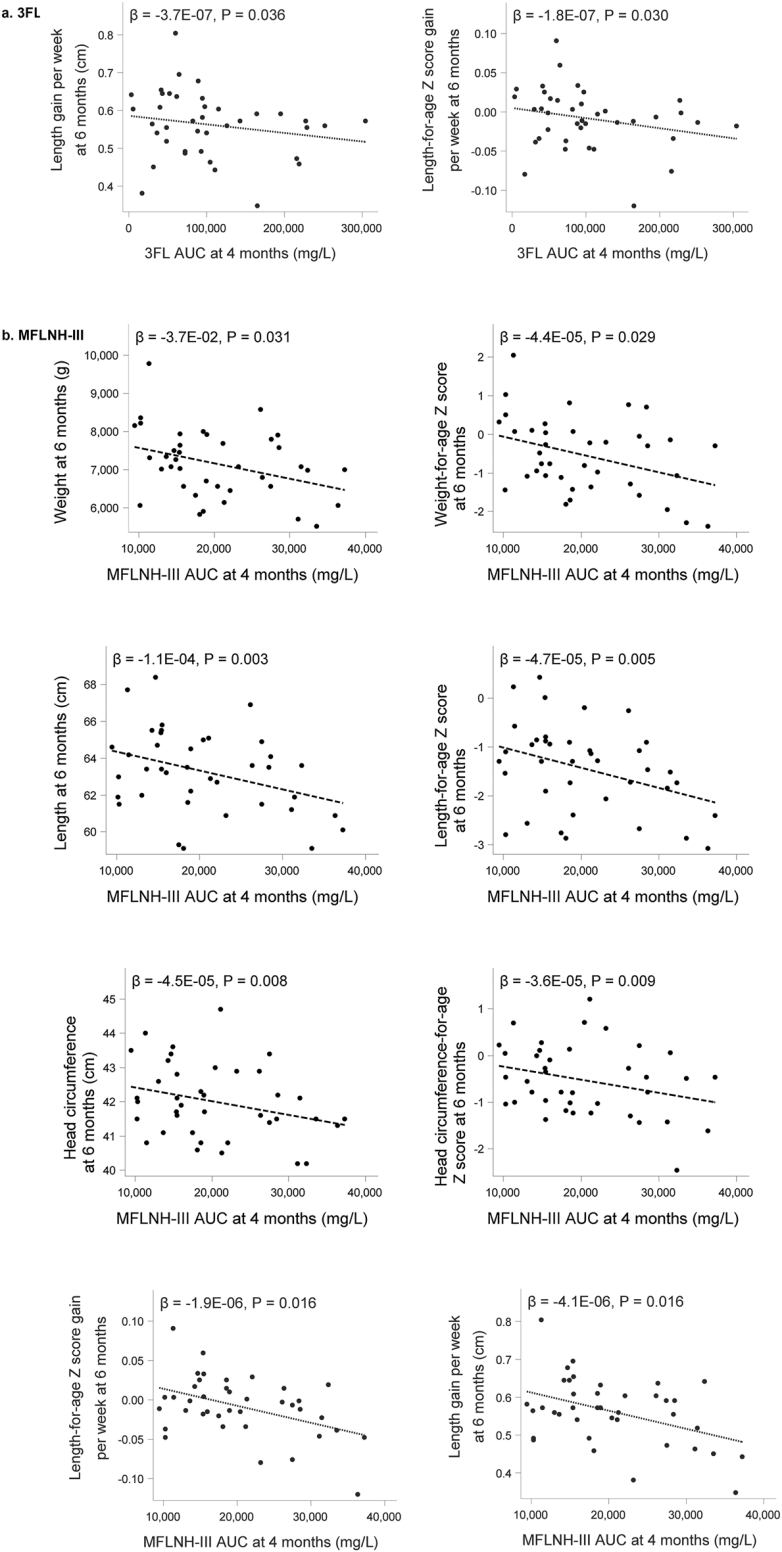
Scatterplots for select HMO Area Under the Curve (AUC) until 4 months of lactation and length gain from birth to 6 months of age (**a**) and weight, length, head circumference and length gain at 6 months and length gain from birth to 6 months of age (**b**) (N = 41). 3FL, 3-fucosyllactose; MFLNH-III, monofucosyllacto-N-hexaose-III.

Seven HMO AUCs had at least one significant association with a growth measure, of which LDFT, LNDFH-I, LNnDFH, 2’FL and 6’GL showed a positive and MFLNH-III and 3FL showed an inverse relation with growth parameters. Scatterplots overlaid with linear regression models for significant positive associations between HMO AUC of LDFT, LNDFH-I, LNnDFH, 2’FL and 6’GL through 4 months and growth measures at 6 months are presented in Fig. 3, while those for the inverse associations with 3FL and MFLNH-III are depicted in Fig. 4. Greater head circumference and head circumference-for age z-scores at 6 months of age were associated with higher AUC for LDFT, LNDFH-I, and LNnDFH over the first 4 months of lactation. LNnDFH AUC up to 4 months of lactation was also positively associated with length and length-for age z-score at 6 months of age. Positive associations between length gain and length-for-age gain z-score through 6 months of age with 2’FL AUC through 4 months of lactation were observed. A similar observation was made with 6’GL and weight gain and weight-for age gain z-score. As shown in Fig. 4a, 3FL AUC through 4 months showed an inverse relation with length gain and length-for age gain z-score through 6 months. Most associations were seen with MFLNH-III AUC through 4 months of lactation (Fig. 4b). MFLNH-III AUC was inversely associated with head circumference and head circumference z-score, weight and weight-for-age z-score, length and length-for-age z-score, all at 6 months, and length gain and length-for-age gain z-score through 6 months of age. Descriptive statistics for growth measures across tertiles of HMO AUCs are shown in Supplemental Table 4. Generally, there were few significant differences in the growth measures across HMO AUC tertiles within females and males; for LNnDFH, longer length was observed in females over higher tertiles (medians in low, medium, and high tertiles 60.1, 63.5, and 61.9 cm, respectively, p = 0.040) and length-for-age z-scores increased over the tertiles (median z-scores − 2.41, − 0.90, and − 1.52, *p* = 0.040). For 3FL, mean length gain/week decreased over the tertiles in males (medians 0.65, 0.63, and 0.57, *p* = 0.026) as did mean length-for-age gain/week z-scores (medians 0.02, 0.01, and − 0.013, *p* = 0.022).

**Table 2:** Descriptive statistics for Baby Eating Behaviour Questionnaire (BEBQ) subscales by infant age (N = 41).

BEBQ Domain	Age of infant					
	22–26 days (N = 41)	42–47 days (N = 41)	2.5 months (N = 41)	2.75 months (N = 40)	4 months (N = 39)	6 months (N = 39)
Food responsiveness	3.6 [0.6] 3.7 (3.2; 4.2)	3.5 [0.8] 3.5 (3.0; 4.2)	3.5 (0.7) 3.5 (3.0; 4.0)	3.6 [0.6] 3.6 (3.2; 4.0)	3.6 [0.8] 3.5 (2.8; 4.3)	3.6 [0.7] 3.7 (3.0; 4.0)
Enjoyment of food	4.8 [0.3] 5.0 (4.8; 5.0)	4.8 [0.4] 5.0 (4.8; 5.0)	4.8 (0.3) 5.0 (4.5; 5.0)	4.8 [0.4] 5.0 (4.8; 5.0)	4.7 [0.4] 5.0 (4.5; 5.0)	4.7 [0.3] 4.8 (4.5; 5.0)
Satiety responsiveness	2.5 [0.8] 2.3 (2.0; 3.0)	2.3 [0.8] 2.3 (1.7; 3.0)	2.3 (0.9) 2.3 (2.0; 2.7)	2.4 [0.7] 2.3 (2.0; 2.7)	2.4 [0.8] 2.3 (2.0; 3.0)	2.4 [0.9] 2.3 (2.0; 3.0)
Slowness in eating	2.5 [0.6] 2.5 (2.0; 2.8)	2.5 [0.6] 2.8 (2.3; 2.8)	2.5 (0.5) 2.5 (2.3; 2.8)	2.6 [0.5] 2.8 (2.3; 3.0)	2.6 [0.6] 2.5 (2.3; 3.0)	2.7 [0.5] 2.8 (2.3; 3.0)
General appetite	5.0 [0.2] 5.0 (5.0; 5.0)	4.9 [0.3] 5.0 (5.0; 5.0)	5.0 (0.2) 5.0 (5.0; 5.0)	4.8 [0.5] 5.0 (5.0; 5.0)	4.8 [0.4] 5.0 (5.0; 5.0)	4.7 [0.6] 5.0 (5.0; 5.0)

Data shown as Mean [Standard Deviation]. Median (25th percentile; 75th percentile).

Of particular interest in view of the associations with growth parameters, we observed the highest concentrations for LDFT and LNDFH-I and the lowest concentrations for MFLNH-III in the Se+ /Le+ milk group. As we observed opposite associations of these HMOs with growth measures, mainly head circumference with LDFT and LNDFH-I (positive) and MFLNH-III (negative), we speculate that these results may reflect the association of Se+/Le+ milk group with greater growth measures. Similarly, 2’FL is highest in Se+ /Le− and 3FL is highest in Se−/Le+ and both show opposite associations to body length measures, again indicating that these results may reflect effects of milk groups. Indeed, when comparing the Se+ /Le+ milk group to the combined other milk groups for infant growth parameters using linear regression models corrected for maternal age at birth, infant sex, birth weight and mode of delivery, we observed that Se+/Le+ was related to greater length and length gain at 4 months of age (Supplemental Table 5). For example, for length comparing the infants in the combined other milk group (Se+/Le−, Se−/Le+, Se−/Le−) to Se+/Le+ , we observed a beta estimate of − 1.4 cm (95% CI − 2.53 to − 0.29; *p* = 0.015). Additionally, infants fed Se+ /Le+ HM compared to the other milk groups had a numerically greater but not significant head circumference with a beta estimate for other milk groups of − 0.46 cm (95% CI − 1.006 to 0.086; *p* = 0.096).

### Associations between BEBQ, HMOs, and growth

Descriptive statistics for each of the subscales of the BEBQ are shown in Table 2 at each study visit. Measures generally had little variation over time, and among the subscales, mean scores were highest for General Appetite and Enjoyment of Food, and lowest for Satiety Responsiveness and Slowness in Eating. There were several significant associations between the BEBQ subscales of Satiety- and Food Responsiveness related to growth measures namely, weight, BMI, weight-for-age z-score, weight-for-length z-score, BMI-for-age z-score, weight gain/day and length-for-age gain z-score, respectively as detailed in Table 3. Using linear regression models between AUCs for each of the HMOs with the BEBQ subscales revealed only few significant associations. Specifically, we observed an association between Enjoyment of Food and LNFP-I (Beta estimate = − 2.919e−4, 95% CI − 5.757e−4; − 8.0e−6, *p* = 0.0442), LNFP-II (Beta = 4.120e−4, 95% CI 3.04e−5; 7.936e−4, *p* = 0.0352), and LNFP-V (Beta = 3.6255e−3, 95% CI 4.312e−4; 6.8198e−3, *p* = 0.0273). No other HMOs were significantly associated with any other BEBQ subscales.

**Table 3:** Model estimates from linear regression analyses of Baby Eating Behavior Questionnaire (BEBQ) subscales AUC and infant growth, N = 41.

BEBQ Subscale	Growth measure	Beta estimate	95% CI	*p* value
Satiety Responsiveness	Weight	2.903	0.460; 5.346	0.0213
	BMI	0.007	0.001; 0.012	0.0144
	Weight-for-age z-score	0.003	0.001; 0.006	0.0186
	Weight-for-length z-score	0.004	0.001; 0.007	0.0118
	BMI-for-age z-score	0.004	0.001; 0.008	0.0100
	Weight gain/day	0.018	0.001; 0.034	0.0387
Food Responsiveness	Length-for-age gain/week z-score	0.000184	0.00005272; 0.0003154	0.0075

Models adjusted for maternal age at birth, gender, birthweight, and mode of delivery. BEBQ subscale AUCs computed up to 6 months. Infant growth measured at 6 months.

## Discussion

For the first time, we report here the concentration of 24 structurally distinct HMOs measured longitudinally over the first 4 months of lactation in a cohort of Filipino mothers and their exclusively breastfed infants. Although this cohort was relatively small, our exploratory analysis revealed indications of a possible relation between specific HMOs in breastmilk and exclusively breastfed infant growth measures, mainly head circumference and body length. However, mostly these were weak, yet statistically significant, signals of small effect size calling for cautious interpretation, especially if extrapolated to other populations. Additionally, we observed associations between Satiety- and Food Responsiveness scores and growth parameters. However, we did not see any significant association between HMOs and Satiety- and Food Responsiveness. Furthermore, we did not observe a link between the Enjoyment of Food associated HMOs, LNFP-I, -II and -V with any growth parameter. Hence, it seems unlikely that HMOs may affect infant growth through the modulation of infant appetite. While the data on the HMO profiles add important information to our understanding on HMO variation among different populations, the observed associations with infant growth and appetitive traits are more challenging to interpret, both in view of previously reported data, plausible mechanisms, and eventually clinical significance.

Globally, the 24 HMOs measured here in a Filipino population over the first 4 months are in the range that has been reported in other geographies^20,30,31,41–46^. Yet, slight differences can be observed when comparing average concentrations, which need to be interpreted with caution due to our study’s small sample size and to its higher proportion of non-secretor mothers compared to other geographies. Globally, an estimated average of 80% of mothers are secretor positive^45,47^ but geographic differences exist. In our Filipino cohort we had 73% secretor positive mothers assessed through the expression of 2’FL in their milk, which is in line with the previously reported secretor positive proportion of 72% in the Filipino population^48^. Our cohort of Filipino mothers showed generally lower concentrations in the secretor-positive HMOs like 2’FL, LNFP-I and LNDFH-I compared to a recent comprehensive review on quantitative HMO data across diverse geographies^45^ or observed in a large European cohort^30^. However, median concentrations of these HMOs from our study corresponded closely to those reported in a meta-analysis from another Asian population in China^41^ indicating some ethnic or geographic differences may exist in the expression of specific HMOs. Concentrations of all other HMOs measured in our cohort were similar to those previously observed in a large cohort of European mothers^30^. Equally, in agreement with previous reports^30,31,42,43,45,49,50^, we observed that HMO concentrations generally decreased over time of lactation with 3FL representing a notable exception to this pattern.

Numerous studies examined HMO concentrations and growth measures in infants and reported rather heterogeneous findings making comparisons difficult^20–24,26–28,34,51^. In our study, we observed most associations between individual HMOs, LDFT, LNDFH-I, LNnDFH and MFLNH-III, and infant head circumference with the former 3 showing a positive association. Both LDFT and LNDFH-I are almost only present in Se+ /Le+ milk groups. Another study from Asia did not find any significant growth differences between infants fed Se+ or Se− breastmilk through the first 4 months after birth^28^. Recently, in one European cohort Se+ breastmilk was associated with lower head circumference through the first year of age^35^, while in another European cohort no such association with head circumference was observed^22^. Additionally, in the same European population MFLNH-III showed a positive correlation with head circumference^22^, while we observed here a negative correlation. Such conflicting findings underscore the need for prudent interpretation. In infants, head circumference has long been shown to be a good proxy for brain volume^52–55^, and several studies have reported associations between increased head circumference and improved markers of cognition and school performance in toddlers and older children^56–58^ with a more recent study also establishing a causal association using data from genome wide associations and Mendelian randomization^59^. However, lack of consistency across different cohorts makes observation-based hypothesis generation challenging.

In our study, LNnDFH and 2’FL were positively and 3FL inversely associated with body length measures. As mentioned earlier, the opposing associations that we see with respect to 2’FL and 3FL with body length, may be reflecting the effects of the milk group, since 2’FL is highest in Se+/Le− and 3FL is highest in Se−/Le+ . In a small cohort of Malawian mothers of infants with healthy growth and with severe stunting, 2’FL in breastmilk at 6 months of age was one of three HMOs that were most discriminatory between healthy and stunted infants^23^. Of note, in our study we observed in the highest tertile of 2’FL a length gain velocity z-score numerically closer to the WHO standard median, while a numerically lower, although not statistically different length gain velocity was observed in the lowest tertile of 2’FL (Supplemental Table 4). Possibly, in infants with lower growth rates slightly higher 2’FL concentrations may support growth within the normal ranges. In a North European study, 2’FL, but not 3FL was reported to have a positive association with infant body length/height and weight^26^, while in another European study height-for-age z-score was neither associated with 2’FL nor 3FL^27^.

The infants in our Filipino cohort had growth parameters that were on the lower side of the WHO growth reference medians despite being within the healthy range, which is consistent with other data from the Philipines^60,61^ and other southeast Asian countries^62,63^. In contrast, infants in the US and Europe tend to be on the higher side of the WHO growth reference medians^64^, which may explain some of the differences observed. Differences in growth rates across the globe indicate that geography-specific associations with HM components such as HMOs may exist and highlight the importance of understanding their role and variability in diverse populations. Adding to this, key HMO-utilizing gut microbes that may contribute to the presumed link between HMOs and infant growth parameters may equally vary between different geographies, something future studies should consider.

Collectively, our results show weak associations of modest effect size that require prudent interpretation due to their exploratory nature. Further, with increasing concentrations of HMOs, all growth parameters were within normal ranges and there was no indication that higher HMO concentrations push infants towards excessive growth or overweight. Rather, from our data it may be postulated that HMOs may play a role in bringing head circumference and length closer to the median in a population growing at the lower end of the WHO growth reference, which may indicate some degree of under-nutrition. However, this requires further exploration and confirmation in well powered studies. This may explain differences observed in other studies of infants from the US and Europe who are higher on the growth charts than Filipino infants and where such associations were not observed^20–22,26,27,35^.

Various mechanisms have been postulated regarding how HMOs may impact growth. The effect of HMOs on the infant microbiota is widely hypothesized as a putative mechanism linking HMO consumption and infant growth through different microbiota mediated mechanisms including appetite regulation^36,37^. We examined infant appetitive traits using a questionnaire specifically designed to assess these in infants (BEBQ) but found very few significant associations between HMO concentrations and the BEBQ subscales (Enjoyment of Food) and none of those consistent with previous data. Plows et al.^40^ in a study of Hispanic infant-mother dyads in the US observed several associations between HMOs and BEBQ subscales including negative associations with LNnT and Food Responsiveness at 1 month, and positive associations with LSTc, LNH, and DSLNH and Food Responsiveness at 6 months. Differences in findings could be related to underlying population differences, small sample size in both studies and spurious findings. Nevertheless, the potentially mediating role of infant appetitive traits between HMO consumption and infant growth is an area of research that should be further developed. In our study, particularly Satiety Responsiveness (a measure of an infant’s fullness threshold)^65^ was associated with multiple measures of growth. Similar associations between BEBQ subscales and infant growth were found in other studies conducted in Australia^66^ and the UK^67,68^.

This study includes several strengths such as a longitudinal cohort with repeated evaluation of HMO concentration and infant growth, carefully controlling for several confounding factors and examining infant appetitive traits as a mechanism by which HMOs may affect infant growth. Most importantly, a Filipino population has not been evaluated before in this context and provides valuable information, particularly about HMO trajectories and associations of overall HMO exposure during the breastfeeding period with growth in a population possibly with some degree of under-nutrition when compared to other studies conducted in Western populations. Study limitations include the small sample size, particularly when examining milk groups, lack of data on the volume of milk intake and not being able to correct for that in the associations with growth, lack of data on maternal BMI, integration of infant gut microbiota and no follow up beyond the first 6 months of age to assess long-term effects of HMOs on growth patterns.

In conclusion, within this longitudinal cohort of exclusively breastfed Filipino infants, we saw statistically significant associations between several HMOs and infant growth parameters although these effects were very modest. Their clinical relevance warrants further research, especially in view of discrepancies seen between different studies. Yet, our preliminary findings suggest that specific HMOs, partly as proxy for the Se+/Le+ milk group, may be associated with length and head circumference, shifting those slightly closer to WHO medians. We hypothesize, these findings may be especially relevant in Asian populations such as the Filipino infants examined herein where growth often tends to be closer to the lower end of the WHO reference range and infants are more likely to experience growth faltering, compared to Western populations. Future research should confirm these associations in diverse populations, including those with some degree of under- or malnutrition, and with longer follow-up periods, as well as gut microbiota and digestive health analysis to elucidate mechanisms by which HMOs may affect infant growth in both the short and long term.

## Methods

### Study design and population

This study was a prospective, observational study of breastfed infants conducted from January 2016 to December 2018 at the Asian Hospital and Medical Center, Muntinlupa City, Philippines, in compliance with the Declaration of Helsinki and the International Conference on Harmonization Guidelines for GCP (Good Clinical Practice). The primary objective of the study was to assess stool consistency. Here, we report the exploratory outcome measures on HMO composition and their association to infant growth and eating behavior of a subgroup for whom HM samples were available. Other exploratory study outcome measures, fecal gut health and immune markers and gut microbiota, were also previously published^69^. Prior to enrollment, written informed consent was obtained from the parent(s)/legally accepted representative (hereafter called parents) of the infants. This study was approved by the Asian Hospital Institutional Review Board, Muntinlupa City, Philippines and is registered on ClinicalTrials.gov (NCT03387124).

Major inclusion criteria were: (1) healthy term, singleton birth (37–42 weeks gestation), (2) post-natal age of 21–26 days at enrollment, (3) weight-for-length and head circumference-for-age z-scores between − 3 and + 3 according to the WHO Child Growth Standards at enrollment, and (4) exclusive breastfeeding and infant’s parents decided to continue exclusive breastfeeding up to 6 months of age. Major exclusion criteria included: (1) evidence of major congenital malformation, (2) significant pre-natal and/or serious post-natal disease before enrollment (by medical decision), (3) admission to Neonatal Intensive Care Unit except for admission for jaundice phototherapy, (4) minor parents, or (5) prior participation in another clinical trial since birth.

In-person clinical study visits occurred at visit 1 (infant age 21–26 days, approximately 0.75 months), visit 2 (infant age 42–47 days, approximately 1.5 months), visit 3 (infant age 2.5 months), visit 4 (infant age 2.75 months), visit 5 (infant age 4 months), and visit 6 (infant age 6 months). Parents were advised not to feed any complementary food or any prebiotic and/or probiotic-containing food/supplement before Study Day 155 (approximately 6 months of age).

### Maternal milk samples

At visits 1, 2, 3 and 5, a subgroup of 41 mothers provided HM samples. The visits occurred within one hour of 11:00 am in order to minimize diurnal variation. HM samples were obtained by a hospital grade electric breast pump from the right breast, and milk was collected until the full breast was empty so that the sample was representative of nutrient levels for one feed including foremilk, midmilk and hindmilk.

HM samples were frozen and stored at − 70 °C until shipment to a laboratory for analysis. Aliquots of HM were shipped on dry ice to Neotron Spa in Italy for quantification of the HMOs using the implemented and validated method as published by Austin and Benet^44^ using retention time for HMO identification. Briefly, HMOs were labeled with the fluorescent tag 2-aminobenzamide and quantified using a standard ultra high liquid chromatography (UHPLC) system equipped with a fluorescence detector, a 2-way 10 port high pressure switching valve and 2 columns, a VanGuard BEH amide (1.7 µm, 2.1 × 50 mm; Waters Corp., Milford, USA) and an Acquity BEH Glycan (1.7 µm, 2.1 × 150 mm; Waters Corp.). The HMOs were measured in each HM sample at each time point and included 2′-fucosyllactose (2′FL), 3-fucosyllactose (3FL), Lacto-N-tetraose (LNT), Lacto-N-neotetraose (LNnT), 3′-sialyllactose (3′SL), 6′-sialyllactose (6′SL), Lacto-N-fucopentaose-I (LNFP-I), LNFP-V, Lacto-N-neofucopentaose (LNnFP), 3′-galactosyllactose (3’GL), 6′-galactosyllactose (6’GL), Alpha-Tetrasaccharide (A-Tetra), Difucosyllacto-N-hexaose a (DFLHNa), disialyllacto-N-tetraose (DSLNT), Hexose N-acetylhexosamine 1 and 2 (Hex4 HexNAC 1 and Hex4 HexNAC 2), Lactodifucotetraose (LDFT), lacto-N-difucohexaose-I (LNDFH-I), Lacto-N-fucopentaose-II (LNFP-II), Lacto-N-fucopentaose-III (LNFP-III), lacto-N-neodifucohexaose (LNnDFH), LS-tetrasaccharide b (LSTb), LS-tetrasaccharide c (LSTc), and Monofucosyllacto-N-hexaose-III (MFLNH-III). The following HMOs were quantified with standard curves using authentic high purity standards: 2’FL, 3FL, LNT, LNnT, LNFP-I, 3’SL, 6’SL and A-Tetra (Elicityl SA., Crolles, France). All other HMOs were quantified using maltotriose (Sigma-Aldrich) for calibration. Each batch of samples analyzed had quality control samples included after every 20 samples to verify the method performance. Deviations within ± 15% from expected amounts were allowed.

Milk groups were defined based on the baseline visit (21–26 days of lactation). A cutoff at 25 mg/L for concentrations of 2’FL and LNFP-II was used to define Secretor (Se) and Lewis (Le) status, respectively, leading to 4 milk groups: 1: Se+/Le+; 2: Se−/Le+; 3: Se+/Le−; 4: Se−/Le−.

### Infant growth measures

Infant growth data including weight, length, and head circumference was collected at visits 1, 2, 3, 4, 5 and 6. Infants were weighed without clothing or diaper on a calibrated electronic weighing scale. The weight measure was repeated until reproduced within 10 g, and the two weights were averaged and recorded to the nearest 1 g (g). The scales were calibrated as per manufacturer’s recommendations at least annually. The same scales were used for all infants at all visits. Length was measured while the infant was dressed in light underclothing or a diaper, without shoes or hair ornaments. Length measurements were performed using a standardized length board, repeated until reproduced within 0.5 cm, and the average of the two measurements was recorded to the nearest 0.1 cm. At least two people were present to maintain proper body alignment and full body extension with feet flexed. Infant head circumference was measured over the most prominent part on the back of the head (occiput) and just above the eyebrows using a standard non-elastic plastic-coated measuring tape pulled snugly to compress the hair. The measurement was repeated until reproduced within 0.2 cm (cm) and the average of the two measures was utilized. Weight gain was computed in g/day and length gain was computed in cm/week. Z-scores including weight-for-age, length-for-age, weight-for-length, BMI-for-age, and head circumference-for-age were calculated using the WHO Child Growth Standards as the reference population^70^.

### BEBQ

The Baby Eating Behavior Questionnaire (BEBQ) is a standardized, validated questionnaire to evaluate infant appetitive traits. The BEBQ was developed from an instrument for older children (Children’s Eating Behavior Questionnaire) and includes 18 questions with 17 designed to measure four aspects of infant feeding behavior (enjoyment of food [EF], food responsiveness [FR], slowness in eating [SE], and satiety responsiveness [SR]) and a single question measuring general appetite (GA)^65,66^. The BEBQ was administered by research staff at Study Days 1, 22, 50, 57, 90, and 155. Items are answered on a 5-point scale which are then scored by calculating a mean for each subscale (EF, FR, SE, SR, and GA) with higher scores indicating greater expression of each behavior.

### Statistical analysis

Demographic, infant, and maternal characteristics were summarized at baseline using descriptive statistics overall and within milk groups. The temporal change of the HMO values was summarized using descriptive statistics at each study visit including mean, standard deviation, minimum, and maximum values. Pearson correlation coefficients between pairs of each of the different HMOs were computed. Infant growth measures are shown using box plots (including minimum, first quartile, median, third quartile, and maximum values) overlaid on WHO infant growth curves, stratified by sex. Overall exposure to each HMO was summarized as area under the curve (AUC) from visit 1 (infant age 21–26 days) to visit 5 (4 months of infant age). The AUC for each HMO was then examined in association with each measure of growth at study day 155 (6 months) using partial Pearson correlations and linear regression, both adjusted for maternal age at birth, infant sex, birth weight, and mode of delivery. For those HMO AUCs with significant associations with growth measures (LDFT, LNDFH-I, LNnDFH, 2’FL, MFLNH-III, 3FL, and 6’GL), the growth measures were summarized by tertiles of HMO AUC within strata of sex, and differences across tertiles were compared using the Kruskal–Wallis test. In order to evaluate the effect of the milk group SE+/LE+ on the growth parameters, the growth measures were descriptively summarized per milk group (SE+/LE + vs. all others) and linear regression adjusted for maternal age at birth, infant sex, birthweight, and mode of delivery and the derived milk groups (SE+/LE+ vs. all others) conducted.

No overall control for multiplicity of testing was done in this study. This is due to its exploratory nature namely rounds of analyses followed by interpretation, leading to new statistical analyses. While false discoveries cannot be excluded, we minimized risks by not interpreting the results based solely on the *p* values, but as well interpreting the magnitude of the effects and their clinical plausibility based on descriptive statistics. Additionally, we looked for consistency across the multiple analyses with observations made in other studies. Finally, our observations can only lead to hypothesis that require confirmation in a properly designed experiment.

All statistical analysis were conducted using SAS^®^ 9.4 software (Wallisellen, Switzerland).

## Supplementary Information

Supplementary Information.

## Data Availability

Study data are available from the corresponding author upon request.
