# Supplementary material for: Dynamics of human milk oligosaccharides in early lactation and relation with growth and appetitive traits of Filipino breastfed infants
Source: Sci Rep. 2022 Oct 15;12:17304. doi: 10.1038/s41598-022-22244-7 (PMC9569346; doi:10.1038/s41598-022-22244-7)
Supplement: Supplementary file 1 — Supplementary Information. [file 41598_2022_22244_MOESM1_ESM.docx]

**SUPPLEMENTAL MATERIAL**

**Dynamics of Human Milk Oligosaccharides in early lactation and relation with growth and appetitive traits of Filipino breastfed infants**

Tinu M. Samuel^1^; Mickaël Hartweg^2^; Jowena D. Lebumfacil^3^, Katherine. B. Buluran^4^, Rachel. B. Lawenko^4^; Elvira M. Estorninos^4^; Aristea Binia^5^; Norbert Sprenger^5*^

Affiliations :

1 Nestlé Product Technology Center-Nutrition, Société des Produits Nestlé S.A., 1800 Vevey, Switzerland.

2 Clinical Research Unit, Société des Produits Nestlé SA, 1000 Lausanne, Switzerland

3 Clinical Research Operations, Wyeth Philippines, Inc., Makati City, 1200, Philippines,

4 Asian Hospital & Medical Center, Muntinlupa City, 1780, Philippines

5 Nestlé Institute of Health Sciences, Société des Produits Nestlé SA, 1000 Lausanne, Switzerland

**Supplemental Table 1.**  Demographic, maternal, and infant characteristics by milk groups, N=41.

|  | **Se+/Le+ (N=25)** | | **Se+/Le- (N=5)** | | **Se-/Le+ (N=10)** | | **Se-/Le- (N=1)** | |
| --- | --- | --- | --- | --- | --- | --- | --- | --- |
|  | **Mean [SD] or N (%)** | **Median**  **[25^th^, 75^th^ percentiles]** | **Mean [SD] or N (%)** | **Median**  **[25^th^, 75^th^ percentiles]** | **Mean [SD] or N (%)** | **Median**  **[25^th^, 75^th^ percentiles]** | **Mean [SD] or N (%)** | **Median**  **[25^th^, 75^th^ percentiles]** |
| **Infant Characteristics** |  |  |  |  |  |  |  | |
| Sex |  |  |  |  |  |  |  | |
| Female | 12 (48%) | | 4 (80%) | | 4 (40%) | | 1 (100%) | |
| Male | 13 (52%) | | 1 (20%) | | 6 (60%) | | 0 (0%) | |
| Gestational age at birth (weeks) | 38.6 [1.2] | 39 [38, 40] | 38.8 [1.6] | 38 [38, 40] | 39.3 [1.8] | 38 [38, 40] | 37.0 [-] | 37 [37, 37] |
| Age at enrollment (days) | 23.6 [1.7] | 24 [22, 25] | 24.4 [2.3] | 26 [23, 26] | 24.0 [2.2] | 25 [21, 26] | 26.0 [-] | 26 [26, 26] |
| Birth weight, grams | 3106.0 [297.4] | 3000  [2900, 3330] | 3106.0 [382.5] | 3070  [2800, 3300] | 3015.0 [347.7] | 3100  [2680, 3360] | 3040.0  [-] | 3040  [3040, 3040] |
| Birth length, cm | 50.3 [2.1] | 50 [49, 52] | 49.2 [0.8] | 49 [49, 50] | 49.7 [2.4] | 49.5 [48, 52] | 48 [-] | 48 [48, 48] |
| Weight at enrollment [21-26 days], grams | 3929.6 [425.6] | 3848  [3580, 4145] | 3733.0 [502.0] | 3990  [3625, 4040] | 3875.5 [310.4] | 3986  [3760, 4068] | 3592.5  [-] | 3593  [3593, 3593] |
| Length at enrollment [21-26 days], cm | 51.0 [1.4] | 50.7  [50.0, 51.8] | 50.1 [1.4] | 49.5  [49.4, 50.2] | 51.0 [2.0] | 50.7  [49.0, 52.9] | 49.2 [-] | 49.2  [49.2, 49.2] |
| BMI at enrollment [21-26 days], kg/m^2^ | 15.1 [1.2] | 15.5  [14.1, 15.8] | 14.9 [1.8] | 15.1  [14.7, 16.3] | 14.9 [1.0] | 15.1  [13.9, 15.4] | 14.8 [-] | 14.8  [14.8, 14.8] |
| Head circumference at enrollment [21-26 days], grams | 36.0 [1.0] | 35.9  [35.3, 36.6] | 35.8 [1.2] | 36.0  [35.4, 36.7] | 35.8 [0.8] | 36.0  [34.8, 36.5] | 35.5 [-] | 35.5  [35.5, 35.5] |
| **Maternal characteristics** |  |  |  |  |  |  |  | |
| Delivery type |  |  |  |  |  |  |  | |
| Vaginal | 23 (92%) | | 4 (80%) | | 9 (90%) | | 0 (0%) | |
| Caesarean | 2 (8%) | | 1 (20%) | | 1 (10%) | | 1 (100%) | |
| Maternal age at birth, years | 26.3 [5.1] | 27  [21, 28] | 26.8 [6.2] | 26  [23, 27] | 26.3 [6.7] | 26.5  [22, 28] | 25.0 [-] | 25  [25, 25] |
| Parity | 2.5 [1.3] | 2 [2, 3] | 3.0 [1.6] | 3 [2, 4] | 2.9 [1.6] | 2.5 [2, 4] | 2.0 [-] | 2 [2, 2] |

**Supplemental Table 2.** Descriptive statistics for quantified HMOs independent of milk group and by time of lactation, N=41.

|  |  | **Time of Lactation** | | | |
| --- | --- | --- | --- | --- | --- |
|  |  | **21-26 days** | **42-47 days** | **2.75 months** | **4 months** |
| 2’FL (mg/L) | n/nmiss/nblq  Mean (SD)  Median  25^th^, 75^th^ percentiles  Min, max | 41 / 0 / 3  1839 (1553)  1795  6.5, 3018  0.0, 4992 | 41 / 0 / 5  1985 (1671)  1909  32, 3121  7, 5684 | 40 / 0 / 3  1195 (975)  1222  23, 1814  0.0, 3205 | 39 / 1 / 6  1091(944)  928  16, 1805  0.0, 2978 |
| 3FL (mg/L) | n/nmiss/nblq  Mean (SD)  Median  25^th^, 75^th^ percentiles  Min, max | 41 / 0 / 0  788 (740)  608  297, 998  0.0, 2829 | 41 / 0 / 0  1120 (943)  875  477, 1273  0.0, 3896 | 40 / 0 / 0  1108 (775)  961  532, 1546  70, 3085 | 39 / 1 / 0  1274 (916)  1160  636, 1839  5.7, 3701 |
| 3’GL (mg/L) | n/nmiss/nblq  Mean (SD)  Median  25^th^, 75^th^ percentiles  Min, max | 41 / 0 / 25  8.5 (9.7)  4.0  4.0, 9.8  4.0, 60 | 41 / 0 / 25  8.3 (8.2)  4.0  4.0, 10.0  0.0, 40 | 40 / 0 / 33  6.1 (5.6)  4.0  4.0, 4.0  4.0, 32 | 39 / 1 / 28  4.4 (4.6)  4.0  4.0, 4.0  0.0, 28 |
| 3’SL (mg/L) | n/nmiss/nblq  Mean (SD)  Median  25^th^, 75^th^ percentiles  Min, max | 41 / 0 / 0  164 (58)  158  128, 196  90, 353 | 41 / 0 / 0  169 (51)  157  132, 200  99, 311 | 40 / 0 / 0  122 (28)  121  100, 136  71, 216 | 39 / 1 / 0  118 (31)  117  94, 135  69, 187 |
| 6’GL (mg/L) | n/nmiss/nblq  Mean (SD)  Median  25^th^, 75^th^ percentiles  Min, max | 41 / 0 / 0  42 (21)  36  30, 52  19, 125 | 41 / 0 / 0  36 (19)  32  23, 44  10, 93 | 40 / 0 / 0  21 (12)  18  13, 25  8.3, 70 | 39 / 1 / 2  16 (10)  14  11, 17  0.0, 60 |
| 6’SL (mg/L) | n/nmiss/nblq  Mean (SD)  Median  25^th^, 75^th^ percentiles  Min, max | 41 / 0 / 0  546 (164)  543  437, 626  232, 1017 | 41 / 0 / 0  321 (122)  285  234, 406  141, 676 | 40 / 0 / 0  143 (68)  129  93, 176  64, 369 | 39 / 1 / 0  86 (41)  84  57, 111  22, 207 |
| A-Tetra (mg/L) | n/nmiss/nblq  Mean (SD)  Median  25^th^, 75^th^ percentiles  Min, max | 41 / 0 / 5  6.2 (23)  0.0  0.0, 0.0  0.0, 136 | 41 / 0 / 3  5.2 (19)  0.0  0.0, 0.0  0.0, 118 | 40 / 0 / 2  3.5 (13x)  0.0  0.0, 0.0  0.0, 71 | 39 / 1 / 1  2.2 (9.2)  0.0  0.0, 0.0  0.0, 52 |
| DFLNHa (mg/L) | n/nmiss/nblq  Mean (SD)  Median  25^th^, 75^th^ percentiles  Min, max | 41 / 0 / 5  148 (160)  116  17, 189  0.0, 617 | 41 / 0 / 4  92 (120)  58  17, 112  0.0, 538 | 40 / 0 / 18  36 (48)  17  17, 44  0.0, 187 | 39 / 1 / 19  16 (23)  17  0.0, 17  0.0, 112 |
| DSLNT (mg/L) | n/nmiss/nblq  Mean (SD)  Median  25^th^, 75^th^ percentiles  Min, max | 41 / 0 / 0  385 (145)  346  290, 471  125, 834 | 41 / 0 / 0  263 (80)  261  202, 303  108, 497 | 40 / 0 / 0  146 (66)  121  98, 193  59, 343 | 39 / 1 / 0  120 (45)  113  88, 146  38, 224 |
| Hex4 HexNAC2 (2) (mg/L) | n/nmiss/nblq  Mean (SD)  Median  25^th^, 75^th^ percentiles  Min, max | 41 / 0 / 11  45 (50)  28  8.0, 58  0.0, 215 | 41 / 0 / 7  42 (48)  29  8.0, 53  0.0, 25 | 40 / 0 / 18  24 (34)  8.0  8.0, 32  0.0, 179 | 39 / 1 / 13  24 (33)  8.0  8.0, 25  0.0, 160 |
| Hex4 HexNAc2 (1) (mg/L) | n/nmiss/nblq  Mean (SD)  Median  25^th^, 75^th^ percentiles  Min, max | 41 / 0 / 0  126 (95)  97  60, 154  24, 447 | 41 / 0 / 0  81 (50)  71  48, 88  31, 266 | 40 / 0 / 5  37 (27)  28  20, 48  0.0, 140 | 39 / 1 / 8  28 (26)  22  8.0, 36  0.0, 135 |
| LDFT (mg/L) | n/nmiss/nblq  Mean (SD)  Median  25^th^, 75^th^ percentiles  Min, max | 41 / 0 / 12  149 (154)  119  23, 255  0.0, 689 | 41 / 0 / 9  260 (404)  208  23, 363  0.0, 2551 | 40 / 0 / 10  203 (189)  182  23, 315  0.0, 880 | 39 / 1 / 11  215 (233)  186  23, 318  0.0, 993 |
| LNDFH-I (mg/L) | n/nmiss/nblq  Mean (SD)  Median  25^th^, 75^th^ percentiles  Min, max | 41 / 0 / 2  758 (732)  888  20, 1297  0.0, 2592 | 41 / 0 / 4  715 (645)  843  18, 1220  0.0, 2133 | 40 / 0 / 6  500 (431)  503  7.8, 823  0.0, 1457 | 39 / 1 / 6  396 (386)  350  5.0, 722  0.0, 1256 |
| LNFP-I (mg/L) | n/nmiss/nblq  Mean (SD)  Median  25^th^, 75^th^ percentiles  Min, max | 41 / 0 / 4  981 (1108)  691  14, 1334  0.0, 4433 | 41 / 0 / 2  649 (758)  426  14, 803  0.0, 2981 | 40 / 0 / 0  336 (397)  247  0.0, 510  0.0, 1665 | 39 / 1 / 2  225 (290)  160  0.0, 262  0.0, 1143 |
| LNFP-II (mg/L) | n/nmiss/nblq  Mean (SD)  Median  25^th^, 75^th^ percentiles  Min, max | 41 / 0 / 0  664 (599)  544  191, 910  0.0, 2081 | 41 / 0 / 0  610 (514)  543  158, 870  0.0, 1763 | 40 / 0 / 1  404 (267)  399  200, 505  0.0, 1029 | 39 / 1 / 1  371 (282)  347  135, 509  0.0, 1145 |
| LNFP-III (mg/L) | n/nmiss/nblq  Mean (SD)  Median  25^th^, 75^th^ percentiles  Min, max | 41 / 0 / 2  277 (142)  255  204, 347  0.0, 766 | 41 / 0 / 2  355 (139)  365  261, 443  18, 629 | 40 / 0 / 1  279 (104)  281  218, 339  18, 499 | 39 / 1 / 2  267 (108)  266  217, 346  0.0, 463 |
| LNFP-V (mg/L) | n/nmiss/nblq  Mean (SD)  Median  25^th^, 75^th^ percentiles  Min, max | 41 / 0 / 6  927 (69)  69  43, 141  12, 242 | 41 / 0 / 9  83 (63)  67  35, 111  12, 257 | 40 / 0 / 11  46 (32)  42  12, 65  0.0, 126 | 39 / 1 / 9  40 (31)  40  12, 55  0.0, 125 |
| LNT (mg/L) | n/nmiss/nblq  Mean (SD)  Median  25^th^, 75^th^ percentiles  Min, max | 41 / 0 / 0  1303 (613)  1243  865, 1504  335, 3637 | 41 / 0 / 0  968 (441)  948  721, 1200  210, 2585 | 40 / 0 / 0  548 (348)  462  327, 659  144, 1828 | 39 / 1 / 0  460 (430)  363  204, 578  104, 2388 |
| LNnDFH (mg/L) | n/nmiss/nblq  Mean (SD)  Median  25^th^, 75^th^ percentiles  Min, max | 41 / 0 / 24  17 (33)  14  0.0, 14  0.0, 212 | 41 / 0 / 22  16 (28)  14  0.0, 14  0.0, 174 | 40 / 0 / 26  14 (15)  14  7, 14  0.0, 87 | 39 / 1 / 20  12 (19)  14  0.0, 14  0.0, 113 |
| LNnFP-V (mg/L) | n/nmiss/nblq  Mean (SD)  Median  25^th^, 75^th^ percentiles  Min, max | 41 / 0 / 24  14 (12)  10  10, 19  0.0, 59 | 41 / 0 / 22  19 (12)  10  10, 28  0.0, 51 | 40 / 0 / 28  14 (12)  10  10, 15  0.0, 59 | 39 / 1 / 26  14 (15)  10  10, 10  0.0, 77 |
| LNnT (mg/L) | n/nmiss/nblq  Mean (SD)  Median  25^th^, 75^th^ percentiles  Min, max | 41 / 0 / 0  226 (157)  191  111, 296  33, 670 | 41 / 0 / 0  243 (153)  211  116, 360  37, 631 | 40 / 0 / 4  153 (125)  107  68, 214  12, 542 | 39 / 1 / 5  128 (118)  82  53, 175  12, 534 |
| LSTb (mg/L) | n/nmiss/nblq  Mean (SD)  Median  25^th^, 75^th^ percentiles  Min, max | 41 / 0 / 0  86 (33)  76  60, 101  21, 165 | 41 / 0 / 0  81 (26)  85  59, 96  17, 143 | 40 / 0 / 0  53 (22)  48  35, 69  17, 108 | 39 / 1 / 0  41 (18)  40  26, 53  15, 95 |
| LSTc (mg/L) | n/nmiss/nblq  Mean (SD)  Median  25^th^, 75^th^ percentiles  Min, max | 41 / 0 / 0  211 (107)  185  150, 242  52, 508 | 41 / 0 / 0  123 (74)  103  67, 159  28, 351 | 40 / 0 / 0  54 (40)  44  22, 74  8.1, 206 | 39 / 1 / 0  31 (23)  24  13, 38  8.9, 110 |
| MFLNH-III (mg/L) | n/nmiss/nblq  Mean (SD)  Median  25^th^, 75^th^ percentiles  Min, max | 41 / 0 / 0  477 (165)  460  376, 555  202, 1017 | 41 / 0 / 0  337 (156)  319  214, 403  89, 662 | 40 / 0 / 0  133 (74)  117  72, 172  39, 332 | 39 / 1 / 9  84 (59)  75  35, 131  18, 246 |

**Supplemental Table 3.** Descriptive statistics for anthropometric measures, by study visit and sex, N=41.

|  | **Mean [SD]**  **Median**  **25^th^, 75^th^ percentiles** | | | | | |
| --- | --- | --- | --- | --- | --- | --- |
| **Infant age at study visit** | **21-26 days** | **42-47 days** | **2.5 months** | **2.75 months** | **4 months** | **6 months** |
|  |  | **BOYS (N=20)** | | | | |
| Weight, g | 3914.4 (487.28)  3971.3  3467.5, 4208.8 | 4881.6 (530.26)  4908.8  4383.8, 5262.5 | 5720.6 (550.67)  5705.0  5297.5, 6170.0 | 5840.5 (531.53)  5853.8  5498.8, 6290.0 | 6566.6 (641.23)  6727.5  6035.0, 7012.5 | 7521.5 (828.94)  7540.0  6995.0, 7932.5 |
| Length, cm | 51.263 (1.773)  50.900  49.650, 53.050 | 53.588 (1.920)  53.250  51.975, 55.150 | 56.920 (2.172)  56.600  55.100, 58.500 | 57.710 (1.903)  57.750  56.550, 58.850 | 60.435 (1.842)  60.650  59.200, 61.500 | 64.320 (2.056)  64.550  62.800, 65.500 |
| Head circumference, cm | 36.085 (1.087)  36.100  35.250, 36.750 | 37.510 (1.074)  37.400  36.725, 38.400 | 39.033 (1.117)  38.900  38.100, 39.900 | 39.353 (1.027)  39.250  38.650, 40.150 | 40.633 (1.076)  40.550  39.775, 41.500 | 42.520 (1.087)  42.400  41.650, 43.400 |
| BMI, kg/m^2^ | 14.855 (1.300)  14.890  13.944, 15.594 | 16.972 (1.306)  16.688  15.922, 18.079 | 17.642 (1.223)  17.581  16.861, 18.144 | 17.518 (1.135)  17.438  16.906, 18.066 | 17.956 (1.328)  17.996  17.333, 18.684 | 18.168 (1.677)  17.971  17.040, 18.833 |
| Weight-for-age z-score | -0.523 (0.872)  -0.550  -1.180, 0.075 | -0.280 (0.802)  -0.200  -0.965, 0.255 | -0.307 (0.796)  -0.300  -0.865, 0.345 | -0.399 (0.760)  -0.365  -0.840, 0.245 | -0.435 (0.8878  -0.155  -1.080, 0.155 | -0.457 (0.979)  -0.385  -1.075, 0.060 |
| Weight-for-length z-score | 0.842 (0.965)  0.880  0.375, 1.450 | 1.726 (0.928)  1.525  1.110, 2.635 | 1.333 (0.926)  1.290  0.450, 1.790 | 1.066 (0.812)  1.065  0.520, 1.250 | 0.853 (0.846)  0.855  0.500, 1.280 | 0.650 (1.064)  0.525  -0.065, 1.075 |
| Length-for-age z-score | -1.309 (0.900)  -1.375  -2.075, -0.460 | -1.529 (0.946)  -1.655  -2.335, -0.605 | -1.372 (1.038)  -1.430  -2.325, -0.540 | -1.325 (0.908)  -1.390  -1.885, -0.710 | -1.418 (0.891)  -1.290  -2.020, -0.800 | -1.426 (0.967)  -1.300  -2.155, -0.865 |
| BMI-for-age z-score | 0.277 (0.941)  0.315  -0.395, 0.895 | 0.831 (0.858)  0.680  0.205, 1.550 | 0.679 (0.806)  0.695  0.125, 1.040 | 0.509 (0.754)  0.450  0.105, 0.910 | 0.540 (0.885)  0.605  0.160, 1.050 | 0.509 (1.073)  0.430  -0.215, 0.995 |
| Head circumference-for-age z-score | -0.574 (0.931)  -0.525  -1.200, 0.045 | -0.627 (0.904)  -0.670  -1.300, 0.075 | -0.585 (0.958)  -0.670  -1.320, 0.170 | -0.587 (0.872)  -0.685  -1.175, 0.075 | -0.643 (0.911)  -0.690  -1.425, 0.100 | -0.571 (0.892)  -0.665  -1.260, 0.160 |
| Weight gain/day | n/a | 45.594 (10.331)  47.030  40.089, 50.595 | 36.691 (6.697)  37.347  35.026, 41.046 | 34.131 (6.116)  34.728  30.938, 38.181 | 29.105 (6.957)  28.530  24.794, 33.915 | 23.423 (5.666)  21.826  20.154, 26.583 |
| Length gain/week | n/a | 0.766 (0.330)  0.758  0.517, 0.969 | 0.803 (0.217)  0.861  0.721, 0.938 | 0.799 (0.177)  0.838  0.756, 0.919 | 0.705 (0.138)  0.719  0.677, 0.773 | 0.594 (0.091)  0.598  0.566, 0.643 |
|  |  | **GIRLS (N=21)** | | | | |
| Weight, g | 3855.5 (310.37)  3847.5  3625.0, 4072.5 | 4668.6 (402.64)  4655.0  4437.5, 4970.0 | 5349.8 (564.42)  5235.0  4895.0, 5555.0 | 5503.9 (598.71)  5395.0  5117.5, 6010.0 | 6108.4 (758.33)  6075.0  5550.0, 6705.0 | 6762.4 (812.4)  6570.0  6060.0, 7350.0 |
| Length, cm | 50.448 (1.267)  50.350  49.400, 51.300 | 52.638 (1.095)  52.500  52.100, 53.150 | 55.581 (1.336)  55.900  54.800, 56.500 | 56.118 (1.372)  56.100  55.625, 56.950 | 59.032 (1.656)  59.400  57.800, 60.400 | 62.205 (1.894)  62.700  60.900, 63.500 |
| Head circumference, cm | 35.690 (0.7409)  35.800  35.000, 36.100 | 36.893 (0.705)  37.000  36.400, 37.450 | 37.983 (0.692)  38.100  37.700, 38.500 | 38.420 (0.499)  38.500  38.050, 38.900 | 39.705 (0.721)  39.800  39.200, 40.100 | 41.468 (0.783)  41.500  40.800, 42.100 |
| BMI, kg/m^2^ | 15.152 (1.126)  15.098  14.658, 15.953 | 16.849 (1.368)  16.918  16.017, 17.778 | 17.302 (1.543)  16.995  16.369, 18.297 | 17.465 (1.655)  17.374  16.149, 18.743 | 17.510 (1.882)  17.248  16.296, 19.170 | 17.453 (1.7262)  17.441  15.790, 19.103 |
| Weight-for-age z-score | -0.183 (0.579)  -0.060  -0.430, 0.150 | -0.036 (0.655)  0.120  -0.370, 0.410 | -0.177 (0.837)  -0.300  -0.740, 0.240 | -0.187 (0.845)  -0.280  -0.690, 0.545 | -0.315 (1.006)  -0.280  -0.980, 0.490 | -0.623 (0.988)  -0.760  -1.440, 0.110 |
| Weight-for-length z-score | 1.162 (0.916)  1.240  0.650, 2.020 | 1.750 (0.925)  1.920  1.200, 2.200 | 1.330 (0.947)  1.200  0.650, 2.000 | 1.308 (1.046)  1.285  0.365, 2.090 | 0.833 (1.156)  0.790  -0.060, 1.800 | 0.509 (1.046)  0.430  -0.520, 1.590 |
| Length-for-age z-score | -1.242 (0.677)  -1.300  -1.800, -0.830 | -1.389 (0.548)  -1.400  -1.660, -1.090 | -1.297 (0.720)  -1.250  -1.570, -0.700 | -1.325 (0.689)  -1.265  -1.610, -0.875 | -1.203 (0.793)  -1.010  -1.820, -0.500 | -1.452 (0.855)  -1.280  -2.070, -0.900 |
| BMI-for-age z-score | 0.703 (0.792)  0.830  0.260, 1.220 | 1.035 (0.885)  1.090  0.580, 1.570 | 0.768 (0.954)  0.580  0.180, 1.400 | 0.779 (1.028)  0.805  -0.030, 1.565 | 0.511 (1.168)  0.400  -0.210, 1.550 | 0.296 (1.079)  0.350  -0.760, 1.340 |
| Head circumference-for-age z-score | -0.327 (0.651)  -0.220  -0.800, 0.140 | -0.462 (0.602)  -0.420  -0.780, -0.040 | -0.681 (0.583)  -0.720  -0.830, -0.210 | -0.556 (0.423)  -0.495  -0.880, -0.190 | -0.525 (0.575)  -0.520  -0.950, -0.240 | -0.482 (0.608)  -0.470  -1.010, 0.040 |
| Weight gain/day | n/a | 38.109 (11.811)  37.738  34.524, 48.333 | 30.293 (10.347)  29.133  23.775, 37.989 | 29.324 (8.860)  28.036  22.835, 37.254 | 24.741 (7.1001  23.187  18.462, 29.753 | 18.867 (4.683)  18.003  15.422, 21.477 |
| Length gain/week | n/a | 0.721 (0.403)  0.800  0.365, 1.013 | 0.728 (0.183)  0.743  0.586, 0.892 | 0.701 (0.166)  0.659  0.581, 0.837 | 0.653 (0.120)  0.646  0.546, 0.754 | 0.530 (0.073)  0.545  0.464, 0.573 |

g = gram; cm = centimeter

**Supplemental Table 4.**  Descriptive statistics for growth parameters by tertiles of HMO area under the curve (AUC), N=41.

| **HMO (mg/L) / Growth Measure** | **HMO AUC: Lowest Tertile** | **HMO AUC: Medium Tertile** | **HMO AUC: Highest Tertile** | ***p-value*** |
| --- | --- | --- | --- | --- |
| **LDFT (mean [SD] concentration)** | **1,939 [1,361]** | **17,633 [6,424]** | **40,859 [16,550]** |  |
| Head circumference | 41.692 [1.000]  41.500 (40.800; 42.600] | 42.400 [1.069]  42.100 (41.600; 43.000) | 41.931 [1.125]  41.900 (41.100; 42.300) | *0.204* |
| Head circ-for-age z-score | -0.878 [0.693]  -0.780 (-1.180; -0.470) | -0.249 [0.817]  -0.270 (-0.960; 0.270) | -0.456 [0.372]  -0.460 (-1.040; 0.040) | *0.160* |
| **LNDFH-I (mean [SD] concentration)** | **779 [720]** | **57,059 [16,314]** | **105,627 [21,280]** |  |
| Head circumference | 41.682 [1.000]  41.500 (40.800; 42.600) | 41.808 [1.152]  41.700 (41.100; 42.100) | 42.523 [0.960]  42.300 (41.700; 43.400) | *0.075* |
| Head circ-for-age z-score | -0.878 [0.693]  -0.780 (-1.180; -0.470) | -0.488 [0.784]  -0.780 (-1.000; 0.040) | -0.217 [0.698]  0.000 (-0.780; 0.230) | *0.097* |
| **LNnDFH (mean [SD] concentration)** | **303 [378]** | **1,255 [68]** | **2,359 [1,249]** |  |
| Length | 62.477 [2.517]  62.000 (60.900; 63.600) | 64.085 [1.909]  64.200 (62.900; 65.000) | 63.308 [2.079]  63.200 (61.900; 64.700) | *0.274* |
| Length-for-age z-score | -1.914 [0.937]  -2.070 (-2.680; -0.900) | -1.045 [0.866]  -1.140 (-1.300; -0.570) | -1.356 [0.728]  -1.300 (-1.540; -1.070) | *0.089* |
| Head circumference | 41.846 [0.945]  41.500 (41.400; 42.800) | 41.854 [1.068]  42.000 (40.800; 42.300) | 42.323 [1.222]  42.100 (41.500; 43.400) | *0.558* |
| Head circ-for-age z-score | -0.770 [0.795]  -0.490 (-1.290; -0.350) | -0.610 [0.703]  -0.96 (-1.04; 0.11) | -0.204 [0.714]  -0.100 (-0.780; 0.210) | *0.153* |
| **2’FL (mean [SD] concentration)** | **9,800 [21,700]** | **127,767 [32,021]** | **250,799 [55,567]** |  |
| Length gain/week | 0.54 [0.085]  0.57 (0.47; 0.59) | 0.57 [0.076]  0.57 (0.54, 0.61) | 0.58 [0.10]  0.56 (0.52; 0.64) | *0.740* |
| Length-for-age z-score gain/week | -0.02 [0.039]  -0.012 (-0.034; -0.001) | -0.00 [0.031]  -0.011 [-0.015; 0.026] | -0.002 [0.043]  0.003 (-0.034; 0.020) | *0.414* |
| **MFLNH-3 (mean [SD] concentration)** | **12,616 [2,212]** | **18,624 [2,193]** | **29,959 [4,149]** |  |
| Weight | 7628.1 [879.76]  7460.0 (7265.0; 8155.0) | 6853.1 [787.66]  6570.0 (6335.0; 7685.0) | 6973.8 [880.27]  6995.0 (6570.0; 7585.0) | *0.070* |
| Weight-for-age z-score | -0.048 [0.945]  0.070 (-0.760; 0.320) | -0.791 [0.771]  -0.970 (-1.360; -0.220) | -0.774 [1.062]  -0.300 (-1.580; -0.140) | *0.093* |
| Length | 64.446 [2.118]  64.600 (63.000; 65.500) | 62.946 [2.052]  63.200 (62.200; 64.500) | 62.477 [2.172]  61.900 (60.900; 63.600) | *0.081* |
| Length-for-age z-score | -1.014 [0.956]  -0.950 (-1.300; -0.570) | -1.484 [0.806]  -1.280 (-1.910; -0.940) | -1.818 [0.813]  -1.740 (-2.410; -1.460) | *0.063* |
| Head circumference | 42.485 [1.031]  42.600 (41.700; 43.400) | 41.792 [1.156]  41.700 (40.800; 42.200) | 41.746 [0.959]  41.500 (41.400; 42.200) | *0.144* |
| Head circ-for-age z-score | -0.238 [0.524]  -0.270 (-0.550; 0.110) | -0.587 [0.820]  -0.960 (-1.180; -0.100) | -0.758 [0.850]  -0.490 (-1.420; -0.280) | *0.140* |
| Length gain/week | 0.604 [0.0878]  0.573 (0.559; 0.645) | 0.569 [0.055]  0.573 (0.541; 0.609) | 0.515 [0.096]  0.518 (0.450; 0.591) | *0.095* |
| Length-for-age z-score gain/week | 0.008 (0.0379)  0.003 (-0.013; 0.025 | -0.001 [0.0228]  0.001 (-0.015; 0.015) | -0.032 [0.041]  -0.023 (-0.047; -0.003) | *0.042* |
| **3 FL (mean [SD] concentration)** | **35,227 [17,492]** | **85,693 [13,739]** | **189,698 [58,298]** |  |
| Length gain/week | 0.586 [0.105]  0.605 (0.541; 0.645) | 0.571 [0.076]  0.573 (0.491, 0.632) | 0.531 [0.076]  0.559 (0.473; 0.573) | *0.304* |
| Length-for-age z-score gain/week | 0.004 [0.0417]  0.004 (-0.023; 0.025) | -0.0003 [0.0319]  -0.011 (-0.020; 0.015) | -0.025 [0.0369]  -0.013 (-0.034; -0.003) | *0.150* |
| **6’GL (mean [SD] concentration)** | **1,462 [321]** | **2,186 [214]** | **3,661 [1,303]** |  |
| Weight-for-length z-score | -0.005 (0.792)  -0.140 (-0.780; 0.430) | 0.7020 [0.826]  0.760 (0.260; 1.270) | 1.029 [1.236]  1.150 (0.090; 2.180) | *0.048* |
| Weight gain/day | 18.661 [3.760]  19.334 (15.422; 20.568) | 22.309 [3.054]  21.477 (20.682; 24.562) | 22.640 [8.1585]  21.023 (16.769; 28.328) | *0.086* |
| Weight-for-age z-score gain/day | -0.004 [0.0045]  -0.004 (-0.006; -0.002) | -0.000363 [0.0041]  -0.000130 (-0.002; 0.003) | 0.001 [0.0093]  -0.002 (-0.004; 0.009] | *0.071* |

Data shown as Mean [Standard Deviation]. Median (25^th^ percentile; 75^th^ percentile). P-value compares mean values of growth measure over tertiles of HMO. Associations presented are those with a significant overall association between the HMO AUC and growth overall.

**Supplemental Table 5.** Descriptive statistics with linear regression model for anthropometric measures by sex and milk group Se+/Le+ (reference) versus other milk groups at 6 months of age, N=41.

|  | **Male** | | | | **Female** | | | | **Linear regression** |
| --- | --- | --- | --- | --- | --- | --- | --- | --- | --- |
|  | **Se+/Le+ (N=13)** | | **Others (N=7)** | | **Se+/Le+ (N=12)** | | **Others (N=9)** | |  |
|  | **Mean [SD]** | **Median**  **[25^th^, 75^th^ percentiles]**  **Min, max** | **Mean [SD]** | **Median**  **[25^th^, 75^th^ percentiles]**  **Min, max** | **Mean [SD]** | **Median**  **[25^th^, 75^th^ percentiles]**  **Min, max** | **Mean [SD]** | **Median**  **[25^th^, 75^th^ percentiles]**  **Min, max** | **Beta estimate**  **95% CI**  **p-value** |
| Weight, g | 7753.5 [850.00] | 7685.0  [7075.0, 8155.0 ]  6705, 9780 | 7090.7 [630.78] | 7010.0  [6570.0, 7640.0]  6060, 7790 | 6898.8 [793.81] | 6827.5  [6237.5, 7405.0]  5700, 8215 | 6528.6 [850.85] | 6460.0  [5835.0, 7085.0]  5515, 7910 | -440.416  -980.108; 99.276  P=0.1063 |
| Length, cm | 64.915 [2.0078] | 64.700  [63.600, 65.800 ]  61.50, 68.40 | 63.214 [1.7686] | 63.600  [61.500, 64.900 ]  60.90, 65.50 | 62.908 [1.6763] | 63.100  [61.900, 63.850]  59.30, 65.40 | 61.000 [1.7098] | 61.000 [1.7098]  [59.100, 62.700]  59.10, 63.50 | -1.408  -2.527; -0.289  P=0.015 |
| Head circumference, cm | 42.808 [1.0120] | 42.900  [42.000, 43.500]  41.60, 44.70 | 41.986 [1.0869] | 42.200  [41.300, 42.800]  40.20, 43.40 | 41.525 [0.8148] | 41.600  [40.950, 42.100]  40.20, 43.00 | 41.371 [0.7783] | 41.500  [40.800, 41.500]  40.60, 42.90 | -0.460  -1.006; 0.086  P=0.0958 |
| BMI, kg/m2 | 18.415 [1.9894] | 18.134  [16.823, 19.542]  16.02, 22.09 | 17.709 [0.7815] | 17.808  [17.256, 18.460]  16.34, 18.49 | 17.420 [1.7727] | 17.583  [15.671, 18.388]  15.22, 20.70 | 17.509 [1.7812] | 16.706  [15.790, 19.366]  15.55, 19.62 | -0.326  -1.534; 0.881  P=0.5861 |
| Weight-for-age z-score | -0.185 [0.9696] | -0.220  [-0.950, 0.320]  -1.42, 2.05 | -0.960 [0.8347] | -1.070  [-1.580, -0.270]  -2.38, -0.05 | -0.439 [0.9384] | -0.450  [-1.235, 0.190]  -1.95, 1.03 | -0.937 [1.0643] | -0.970  [-1.810, -0.210]  -2.28, 0.71 | -0.530  -1.164; 0.104  P=0.0985 |
| Length-for-age z-score | -1.156 [0.9439] | -1.300  [-1.720, -0.790]  -2.80, 0.43 | -1.926 [0.8528] | -1.740  [-2.680, -1.080]  -3.08, -0.88 | -1.121 [0.7455] | -1.025  [-1.530, -0.735]  -2.76, 0.01 | -2.020 [0.7616] | -2.070  [-2.870, -1.280]  -2.87, -0.90 | -0.650  -1.168; -0.132  P=0.0154 |
| Weight-for-length z-score | 0.764 [1.2815] | 0.640  [-0.220, 1.530]  -0.89, 3.08 | 0.439 [0.4670] | 0.410  [0.090, 0.870]  -0.35, 0.88 | 0.439 [1.0833] | 0.525  [-0.655, 1.165]  -0.89, 2.28 | 0.629 [1.0494] | 0.370  [-0.250, 1.710]  -0.68, 1.80 | -0.094  -0.845; 0.658  P=0.8017 |
| Head circumference-for-age z-score | -0.342 [0.8339] | -0.280  [-1.040, 0.230]  -1.37, 1.21 | -0.997 [0.8977] | -0.780  [-1.610, -0.350]  -2.46, 0.21 | -0.424 [0.6332] | -0.365  [-0.890, 0.050]  -1.42, 0.71 | -0.581 [0.5952] | -0.490  -1.030, -0.470  -1.18, 0.58 | -0.376  -0.821; 0.068  P=0.0941 |
| BMI-for-age z-score | 0.652 [1.2690] | 0.540  [-0.370, 1.440]  -0.97, 2.87 | 0.243 [0.5454] | 0.320  [-0.060, 0.760]  -0.73, 0.78 | 0.274 [1.1068] | 0.435  [-0.840, 0.925]  -1.17, 2.21 | 0.333 [1.1145] | -0.130  -0.760, 1.490  -0.93, 1.63 | -0.180  -0.942; 0.583  P=0.6349 |
| Weight gain/day | 24.421 [6.3015] | 21.786  [20.049, 26.802]  19.19, 41.56 | 21.570 [4.0110] | 21.867  [20.568, 24.172]  13.70, 26.49 | 19.697 [4.7717] | 18.701  [15.820, 23.303]  14.06, 29.01 | 17.444 [4.5068] | 16.380  [12.484, 20.682]  12.19, 24.56 | -2.456  -6.105; 1.193  P=0.1802 |
| Length gain/week | 0.623 [0.0748] | 0.632  [0.582, 0.645]  0.49, 0.80 | 0.539 [0.0986] | 0.573  [0.473, 0.591]  0.35, 0.64 | 0.550 [0.0616] | 0.550  [0.505, 0.568]  0.46, 0.70 | 0.495 [0.0821] | 0.459  [0.443, 0.573]  0.38, 0.60 | -0.065  -0.117; -0.013  P=0.0154 |
| Head circumference gain/week | 0.296 [0.0296] | 0.293  [0.280, 0.305]  0.26, 0.37 | 0.287 [0.0416] | 0.286  [0.264, 0.309]  0.22, 0.35 | 0.269 [0.0277] | 0.264  [0.245, 0.290]  0.23, 0.31 | 0.260 [0.0232] | 0.266  [0.245, 0.273]  0.22, 0.30 | -0.010  -0.032; 0.011  P=0.3429 |
| BMI gain/day | 0.021 [0.0159] | 0.016  [0.010, 0.028]  0.00, 0.05 | 0.022 [0.0076] | 0.020  [0.016, 0.031]  0.02, 0.04 | 0.016 [0.0120] | 0.016  [0.008, 0.021]  -0.00, 0.05 | 0.014 [0.0147] | 0.019  [0.001, 0.028]  -0.01, 0.03 | -0.001  -0.010; 0.009  P=0.8984 |
| Weight for age z-score gain | 0.001 [0.0081] | -0.001  [-0.005, 0.006]  -0.01, 0.02 | -0.001 [0.0061] | -0.002  [-0.003, 0.000]  -0.01, 0.01 | -0.002 [0.0059] | -0.003  [-0.007, 0.002]  -0.01, 0.01 | -0.004 [0.0052] | -0.003  [-0.010, 0.000]  -0.01, 0.00 | -0.002  -0.007; 0.002  P=0.2724 |
| Length for age z-score gain | 0.009 [0.0345] | 0.010  [-0.011, 0.025]  -0.05, 0.09 | -0.032 [0.0481] | -0.013  -[0.075, -0.006]  -0.12, 0.02 | -0.005 [0.0282] | -0.008  [-0.021, 0.003]  -0.05, 0.06 | -0.022 [0.0382] | -0.034  [-0.047, 0.015]  -0.08, 0.03 | -0.028  -0.053; -0.004  P=0.0215 |
| Weight for length z-score gain | -0.016 [0.0692] | -0.035  [-0.074, 0.038]  -0.09, 0.11 | 0.004 [0.0479] | -0.018  [-0.031, 0.060]  -0.04, 0.08 | -0.023 [0.0550] | -0.017  [-0.055, -0.008]  -0.10, 0.12 | -0.033 [0.0761] | -0.007  [-0.108, 0.030]  -0.15, 0.06 | 0.005  -0.040; 0.051  P=0.8167 |
| Head circumference for age z-score gain | 0.003 [0.0245] | 0.000  [-0.013, 0.013]  -0.02, 0.06 | -0.006 [0.0351] | -0.003  [-0.031, 0.006]  -0.05, 0.06 | -0.004 [0.0222] | -0.007  [-0.019, 0.014]  -0.04, 0.03 | -0.005 [0.0193] | -0.005  [-0.015, 0.006]  -0.04, 0.02 | -0.006  -0.024; 0.011  P=0.4625 |
| BMI for age z-score gain | 0.001 [0.0105] | -0.002  [-0.007, 0.008]  -0.01, 0.02 | 0.002 [0.0057] | -0.001  [-0.001, 0.008]  -0.00, 0.01 | -0.002 [0.0078] | -0.001  [-0.007, 0.001]  -0.01, 0.02 | -0.003 [0.0095] | 0.002  [-0.011, 0.006]  -0.02, 0.01 | 0.000  -0.006; 0.006  P=0.9529 |

Beta estimate for ‘Other’ milk group compared to Se+/Le+ (reference) group from linear regression model further adjusted for maternal age at birth, infant sex, birth weight, and mode of delivery.

**Supplemental Figure 1.** Pearson Correlation plot for all HMOs.

**Supplemental figure 2.** Infant growth over the first 6 months of life, by gender (N=41) shown as a) infant weight plotted against WHO percentiles, b) infant length plotted against WHO percentiles, c) infant head circumference plotted against WHO percentiles, d) z-scores for weight-for-age, e) z-scores for weight-for-length, f) z-scores for length-for-age, and g) z-scores for head circumference-for-age. Box plots show median with 25^th^ to 75^th^ percentile and whiskers indicate 5^th^ and 95^th^ percentiles.

**a.**


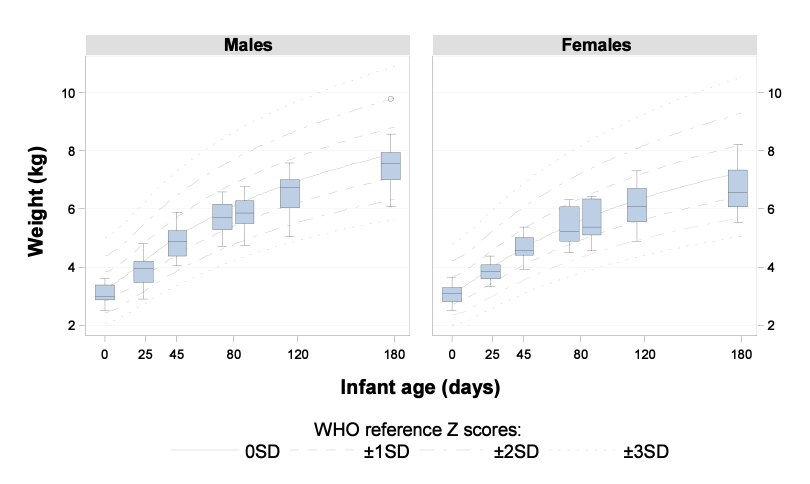


**b.**


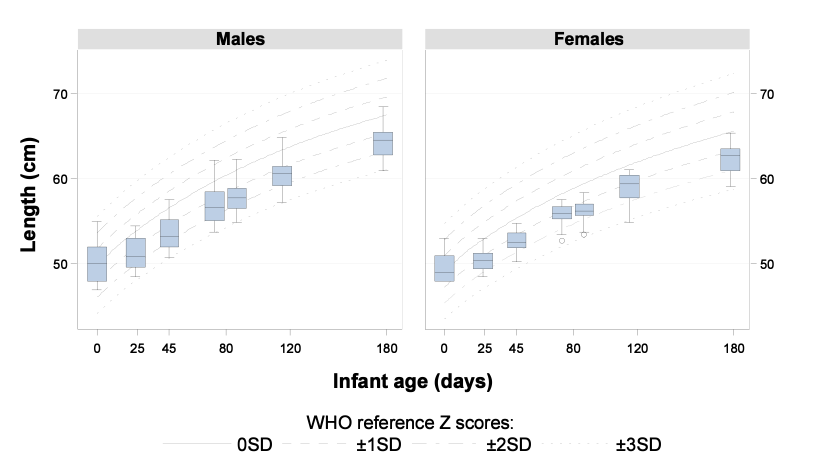


**c.**


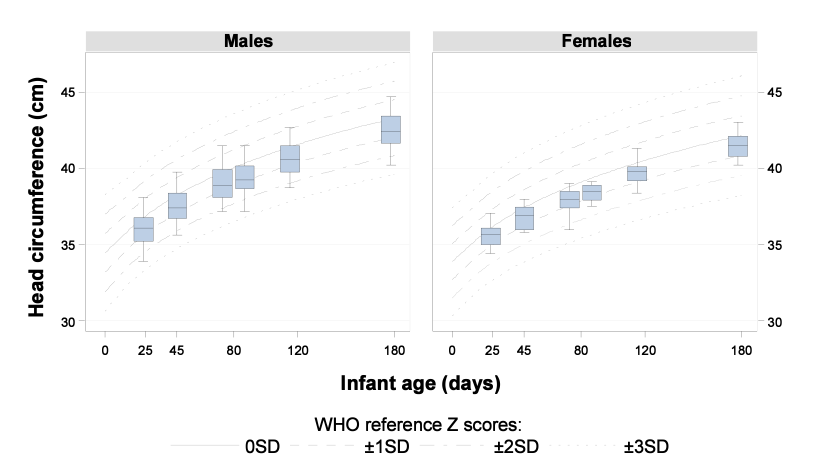


**d.**


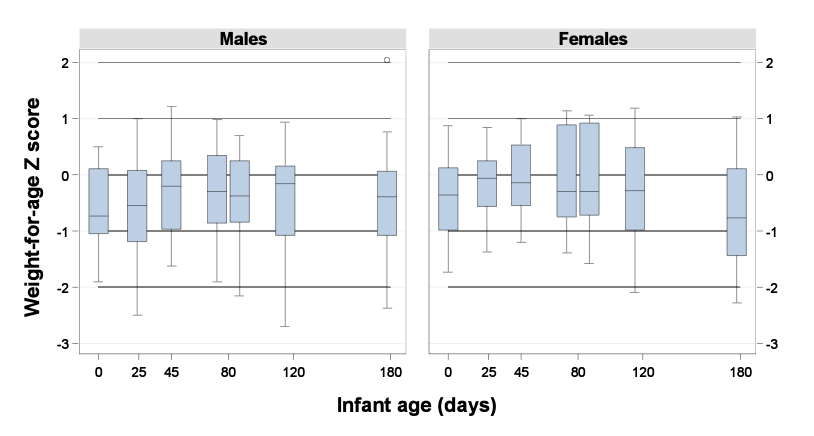


**e.**

**
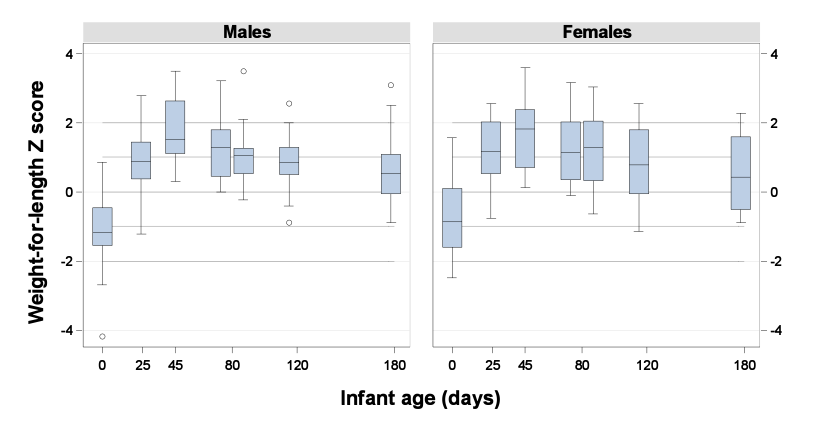
**

**f.**

**
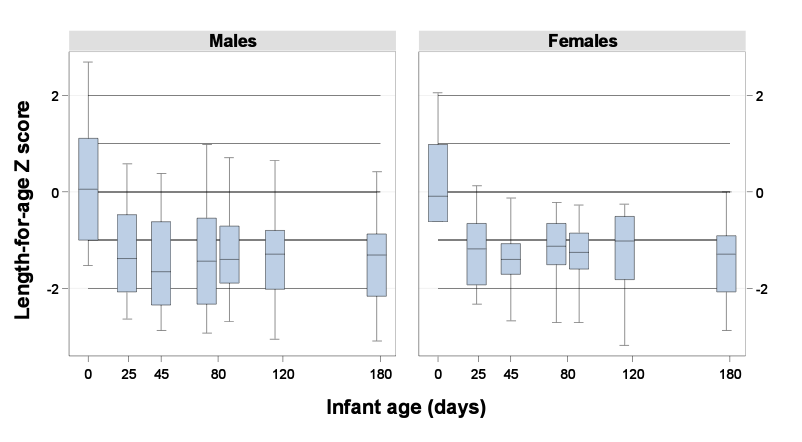
**

**g.**


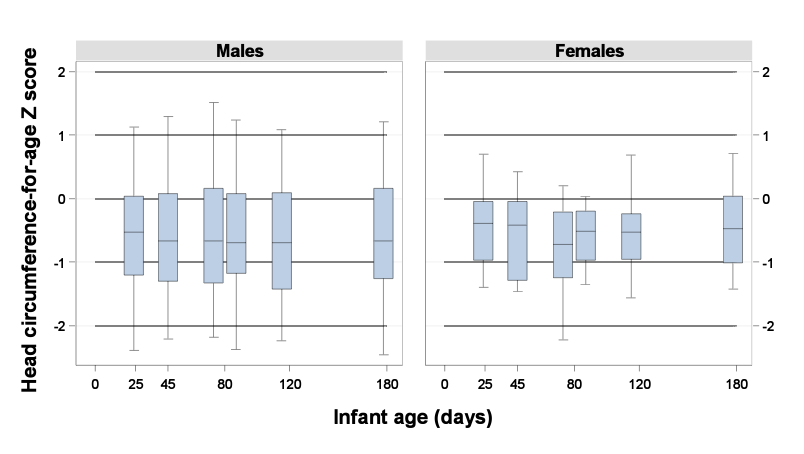


**Supplemental Figure 3.** Infant growth across the first 6 months (N=41), stratified by gender, shown as a) weight gain/day, b) length gain/week, c) head circumference gain/week, d) BMI, e) BMI-for-age z-score, f) BMI gain/day, g) weight z-score gain/day, h) length z-score gain/week, i) weight-for-length z-score gain/week, j) head circumference z-score gain/week, and k) BMI z-score gain/day.

**a.**


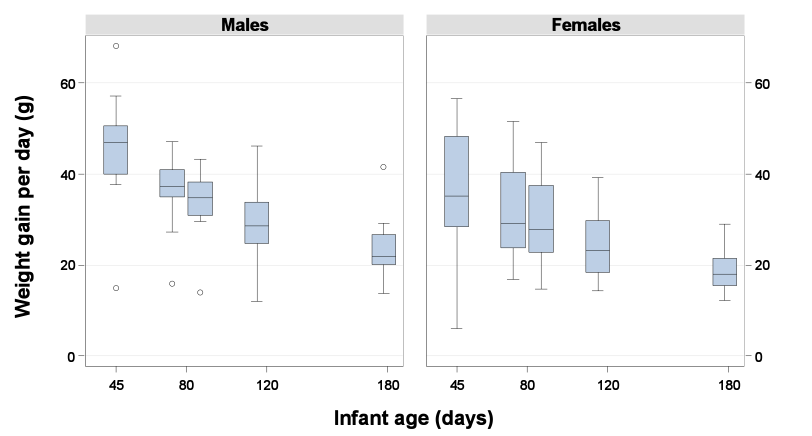


**b.**

**
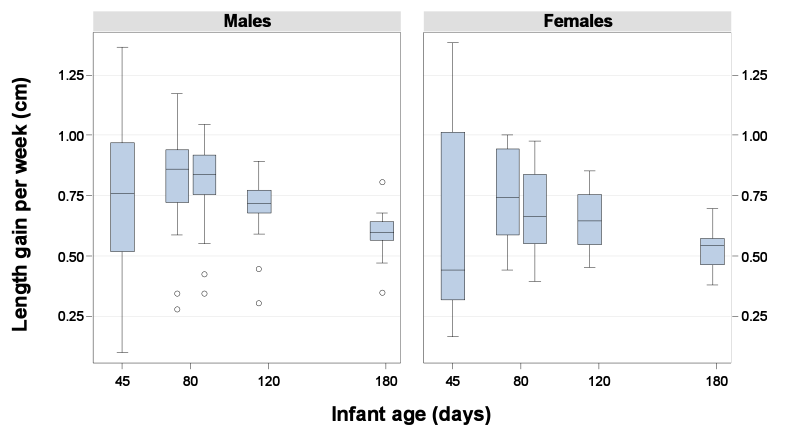
**

**c.**

**
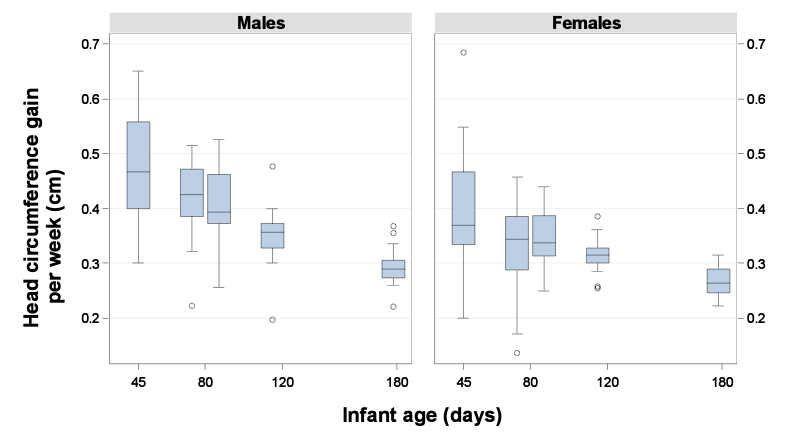
**

**d.**

**
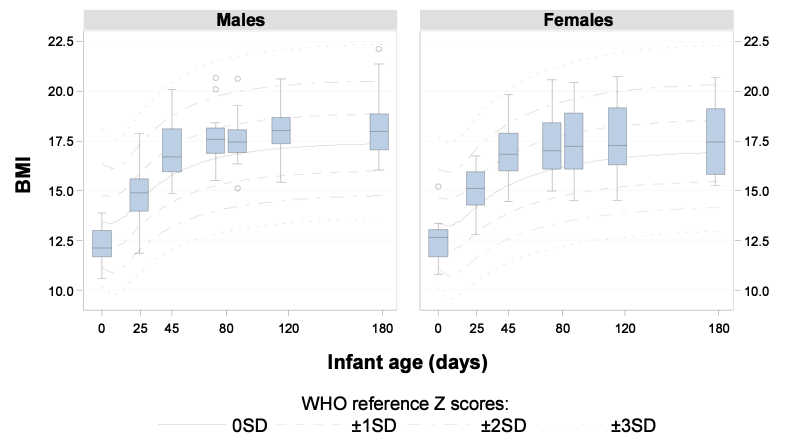
**

**e.**

**
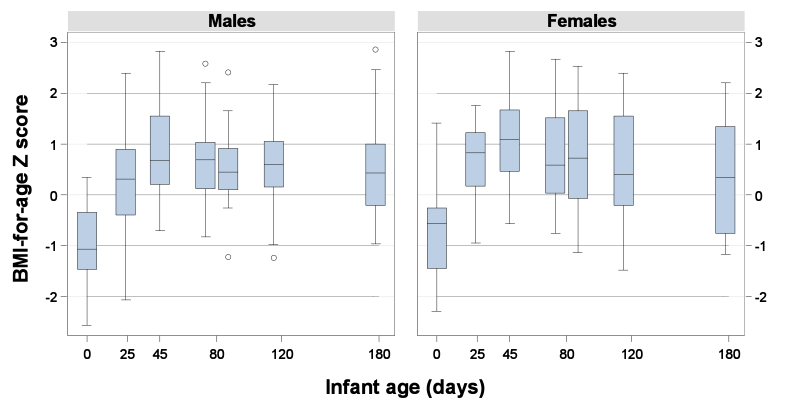
**

**f.**

**
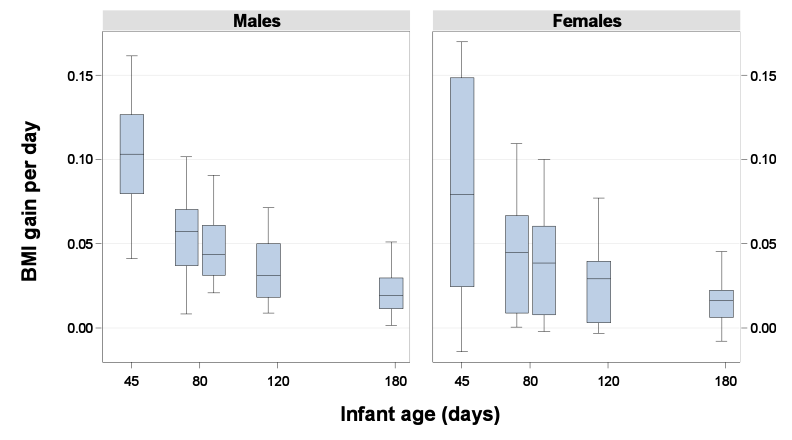
**

**g.**

**
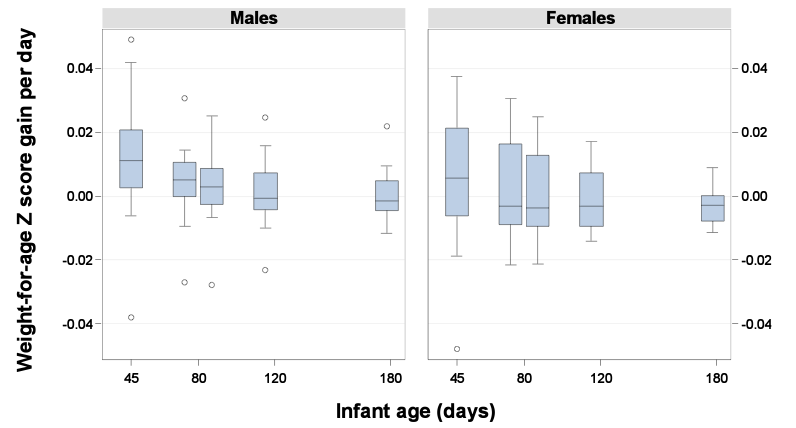
**

**h.**

**
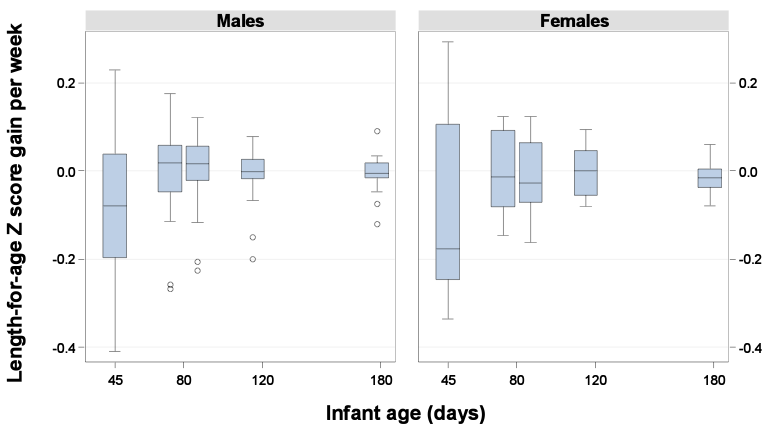
**

**i.**


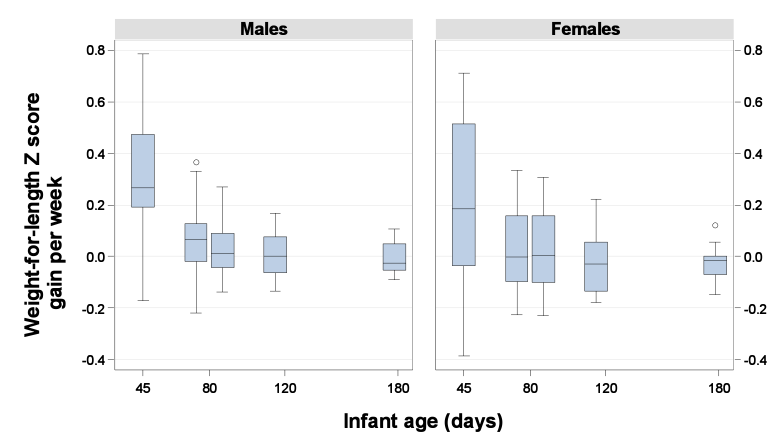


**j.**

**
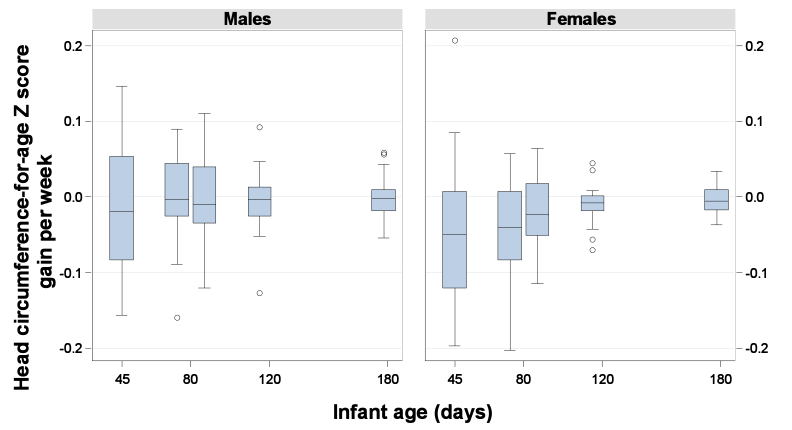
**

**k.**

**
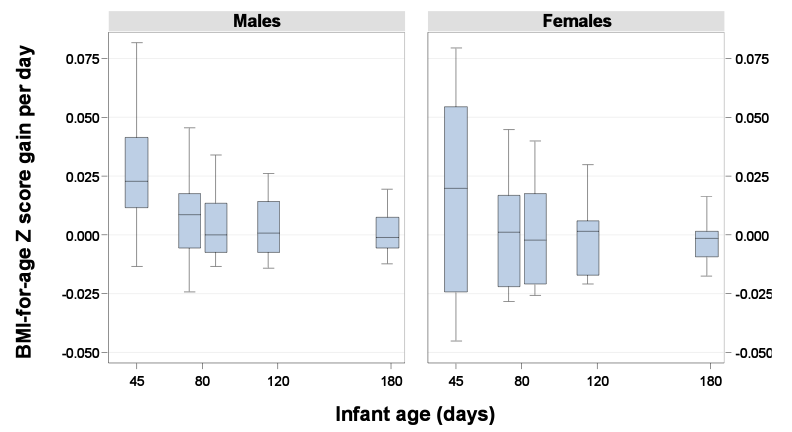
**
